# An Autism-Associated Variant of Epac2 Reveals a Role for Ras/Epac2 Signaling in Controlling Basal Dendrite Maintenance in Mice

**DOI:** 10.1371/journal.pbio.1001350

**Published:** 2012-06-26

**Authors:** Deepak P. Srivastava, Kevin M. Woolfrey, Kelly A. Jones, Charles T. Anderson, Katharine R. Smith, Theron A. Russell, Hyerin Lee, Marina V. Yasvoina, David L. Wokosin, P. Hande Ozdinler, Gordon M. G. Shepherd, Peter Penzes

**Affiliations:** 1 Department of Physiology, Northwestern University Feinberg School of Medicine, Chicago, Illinois, United States of America; 2 Department of Neuroscience & Centre for the Cellular Basis of Behaviour, The James Black Centre, King's College London, Institute of Psychiatry, London, United Kingdom; 3 Weinberg College of Arts and Sciences, Northwestern University, Evanston, Illinois, United States of America; 4 Davee Department of Neurology, Northwestern University Feinberg School of Medicine, Chicago, Illinois, United States of America; 5 Cognitive Neurology and Disease Center, Northwestern University Feinberg School of Medicine, Chicago, Illinois, United States of America; 6 Lurie Cancer Research Center, Northwestern University Feinberg School of Medicine, Chicago, Illinois, United States of America; 7 Department of Psychiatry and Behavioral Sciences, Northwestern University Feinberg School of Medicine, Chicago, Illinois, United States of America; University of Cambridge, United Kingdom

## Abstract

The architecture of dendritic arbors determines circuit connectivity, receptive fields, and computational properties of neurons, and dendritic structure is impaired in several psychiatric disorders. While apical and basal dendritic compartments of pyramidal neurons are functionally specialized and differentially regulated, little is known about mechanisms that selectively maintain basal dendrites. Here we identified a role for the Ras/Epac2 pathway in maintaining basal dendrite complexity of cortical neurons. Epac2 is a guanine nucleotide exchange factor (GEF) for the Ras-like small GTPase Rap, and it is highly enriched in the adult mouse brain. We found that in vivo Epac2 knockdown in layer 2/3 cortical neurons via in utero electroporation reduced basal dendritic architecture, and that Epac2 knockdown in mature cortical neurons in vitro mimicked this effect. Overexpression of an Epac2 rare coding variant, found in human subjects diagnosed with autism, also impaired basal dendritic morphology. This mutation disrupted Epac2's interaction with Ras, and inhibition of Ras selectively interfered with basal dendrite maintenance. Finally, we observed that components of the Ras/Epac2/Rap pathway exhibited differential abundance in the basal versus apical dendritic compartments. These findings define a role for Epac2 in enabling crosstalk between Ras and Rap signaling in maintaining basal dendrite complexity, and exemplify how rare coding variants, in addition to their disease relevance, can provide insight into cellular mechanisms relevant for brain connectivity.

## Abstract 2

Epac2 disruption impairs basal (but not apical) dendrite complexity in cortical neurons, and an autism-associated mutation in Epac2 implicates a Ras/Epac2 signaling pathway in the active maintenance of basal dendritic arbors.

## Author Summary

A fundamental feature of a neuron is the morphology of its dendrites, which are the processes that receive and integrate synaptic signals from other neurons. Neurons in the mammalian cortex exhibit two distinct dendritic arbors: apical dendrites, which extend far from the cell body, and basal dendrites, which elaborate locally around the cell body. After development, neurons must actively maintain each of these dendritic arbors to sustain their specific connectivity. Because several neurological and neurodevelopmental disorders are associated with disruptions in dendritic morphology, it is crucial to understand the molecular mechanisms that regulate the process of active maintenance of dendritic arbors. We find that disruption of a particular molecular pathway, the Ras-Epac2 pathway, can result in dramatic simplification of basal, but not apical, dendritic arbors in both cultured neurons and in the intact mouse brain. We show that a mutant form of Epac2, identified in patients with autism, also impairs basal dendrite maintenance and disrupts its interaction with Ras. Our findings suggest that specific molecular pathways can regulate distinct dendritic regions, and that disease-related mutations can inform our understanding of the molecules that regulate important biological processes.

## Introduction

Dendritic structure is critical for neuronal function, as the size and shape of the dendritic arbor defines the neuron's receptive field [1]. Generating and maintaining proper arborization is therefore crucial for neural circuit function [2]. The importance of maintaining dendritic arborization is illustrated by observations of loss of dendritic complexity in patients with neuropsychiatric disorders. Reduced dendritic arborization occurs in patients with psychiatric disorders with delayed onset, including schizophrenia [3], as well as in autism spectrum disorders [4],[5], and disorders comorbid with autism, such as Rett [6] and Down syndromes [7]–[9]. Thus, the maintenance of dendritic arbor complexity for extended periods of time during development and into adulthood is likely to be crucial for the preservation of functional circuitry and connectivity relevant for learning and complex behaviors.

Patterns of dendritic branching are integral to the computational ability of the neuron [10],[11]. An increasing body of evidence suggests that the apical versus basal regions of the dendritic arbor are functionally specialized. These distinct dendritic compartments receive different inputs, integrate distinct signals, and are selectively regulated in physiological and pathological conditions [12],[13]. Recent work probing the subcellular location of thalamocortical and intracortical connections has revealed tight spatial restriction of synapses to various somatic and dendritic compartments. Laminar positioning of target cells in the cortex and afferent cell type are critical determinants of synaptic positioning along the dendritic arbor. For example, ascending inputs target basal dendrites in layer 2/3 [13]. Basal dendrites are also the target of substantial inhibitory innervation by interneurons, allowing for the tight regulation of excitability [14]. From a computational perspective, even very small basal dendrites are capable of large effects on cell output [12]. Thus, subtle morphological alterations to the basal dendritic arbor may have large consequences for cellular and circuit function.

Consistent with the selective function of dendritic compartments, there is evidence for the selective regulation and maintenance of apical versus basal dendritic compartments. Environmental enrichment appears to have region- and cell-specific effects on dendrites, but preferentially enhances basal arborization [15],[16], and sensory deprivation during a critical developmental period can prevent normal basal dendritic elaboration in the barrel cortex of rats [17]. While a few molecular alterations selectively affect distinct dendritic compartments, including PTEN or dopamine receptor D1 loss [18],[19], the molecular mechanisms that specifically govern basal dendrite maintenance in cortical neurons remain unclear.

Regulators of Ras-like small GTPases have been extensively implicated in neuronal morphogenesis [20]. The EPAC2 gene encodes Epac2 (exchange protein directly activated by cyclic AMP 2), a guanine nucleotide exchange factor (GEF) for the Ras-like small GTPase Rap, which is highly enriched in the adult brain [21] and dendrites [22]. Previous studies utilizing an Epac-specific agonist have found that Epac activation can modulate synaptic plasticity [23] as well as memory retrieval in mice [24], and EPAC null mice exhibit deficits in spatial reference memory and social interactions [25], implicating Epac in brain function. Epac2 has been implicated in the outgrowth of neuronal processes in vitro [26],[27], but its role in dendritic morphogenesis within the cortex is not known. In the present study we observed that Epac2 knockdown robustly and selectively impaired basal dendrite maintenance in cortical pyramidal neurons in vivo and in culture.

Recent genetic studies have detected numerous rare coding mutations in subjects with neurodevelopmental disorders [28]. While their significance for disease etiology remains to be elucidated, such mutations might provide insight into a protein's functional role in important cellular processes. Previously, four rare amino acid coding variants had been identified in EPAC2 in subjects with autism [29]. In the present study we observed that expressing one of these rare coding variants robustly and selectively reduced basal dendrite complexity in cortical pyramidal neurons and impaired Epac2's interaction with Ras. The use of a disease-associated point mutation as a method of probing molecular function revealed that Epac2 mediates crosstalk between Ras and Rap to specifically regulate basal dendritic complexity in cortical neurons. This approach exemplifies a more general “reverse translational” strategy for discovery of basic cellular mechanisms.

## Results

### Epac2 Is Selectively Required for the Maintenance of Basal Dendrite Complexity In Vivo

**Figure 1:**
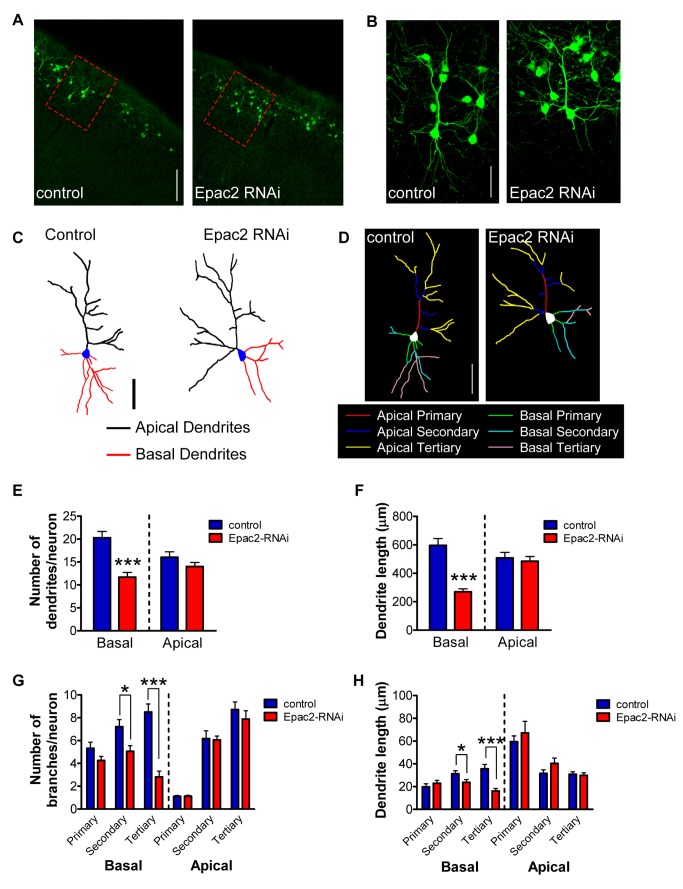
Epac2 regulates basal dendrite complexity in vivo. (A) Low magnification images of in utero electroporated layer 2/3 cortical neurons, expressing either control or Epac2-RNAi. Animals were electroporated at E16.5; coronal slices (50 µm) were made at P28. Red dashed rectangles indicate images represented in (B–D). (B) High magnification images of layer 2/3 cortical neurons outlined in (A). (C–D) Binary images (C) and “skeleton outline” of basal and apical arbors (D), separated into primary, secondary, and tertiary branches, of neurons shown in (B). (E) Quantification of dendrite numbers separated into total apical and basal dendrite branches. Epac2 knockdown in vivo induced a loss of basal dendrites. (F) Epac2 knockdown in vivo induced a selective reduction in basal dendrite length. (G) Quantification of dendrite branch numbers separated into basal/apical, primary/secondary/tertiary order branches. Epac2 knockdown in vivo induced a loss of basal dendrites, specifically driven by a loss of secondary and tertiary basal dendrites. (H) Epac2 knockdown in vivo selectively reduced secondary and tertiary basal dendrite lengths. *p<0.05, ***p<0.001; scale bars, 100 µm (A); 50 µm (B–D).

Epac2, an upstream regulator of Rap activity, is involved in regulating synapse morphology [22], but its role in regulating the architecture of the dendritic tree in cortical neurons is unknown. We first tested whether altered expression of Epac2 affected the maintenance of dendritic arbors in vivo, using in utero electroporation (IUEP) [30] to knock down protein expression (Figure S1A). Using a previously characterized RNA interference (RNAi) construct selective for Epac2 [22], we coinjected either Epac2-RNAi, or control (pGSuper), with pCAG-eGFP into the subventricular zone of E16.5 mouse embryos to specifically target layer 2/3 neurons. Electroporated constructs were allowed to express until brain harvesting and sectioning on postnatal day 28 (P28). In vivo knockdown of the Epac2 protein via Epac2-RNAi was confirmed at P28 by immunohistochemistry of cortical sections. Quantification of GFP-positive cells revealed that Epac2 was knocked down by ∼75% compared to GFP-negative cells (Figure S1B–C). Electroporated neurons were specifically found in layer 2/3 (Figure 1A). When we analyzed dendritic morphology by measuring apical and basal dendritic number and length in GFP-positive cells, we observed that long-term knockdown of Epac2 expression specifically reduced basal dendrites. Examination of basal arbors of layer 2/3 neurons from P28 mice expressing GFP and Epac2-RNAi (Figure 1B–F) revealed a ∼42% reduction in basal dendritic branches and a ∼54% reduction in basal dendritic length (*p*<0.001; Figure 1E–F) compared to cells from P28 mice expressing GFP and control construct. This effect on basal dendritic morphology was mediated by a ∼30% reduction in secondary and a ∼65% reduction in tertiary order basal branch number (*p*<0.05, 0.001; Figure 1G, Table S1A). Additionally, basal dendritic length was reduced by ∼24% in secondary branches and ∼55% in tertiary branches (*p*<0.05, 0.001; Figure 1H, Table S1B). The effect of Epac2 knockdown from E16.5 to P28 was specific for basal dendritic arbors, as Epac2-RNAi had no effect on apical dendrite branch number or length in these cells (Figure 1E–H, Table S1A–B).

**Figure 2:**
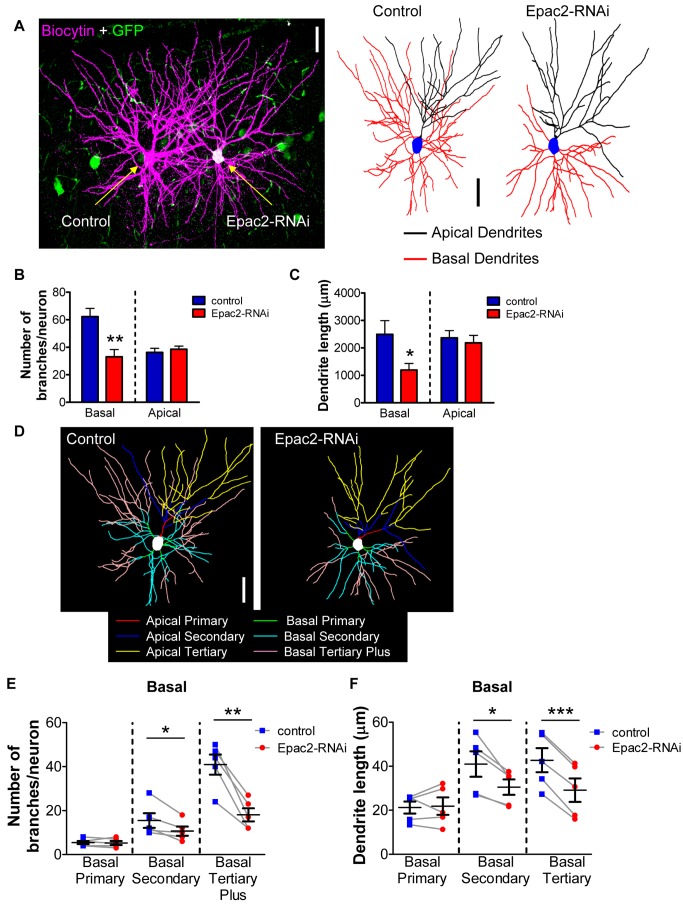
Comparison of paired cell morphology following in vivo knockdown of Epac2 by in utero electroporation reveals reduced basal arbors of layer 2/3 cortical neurons. (A) Left, image of paired cells, one positive for Epac2-RNAi, filled with biocytin and imaged by 2PLSM in 300 µm-thick cortical slices. Right, binary images of biocytin-filled paired layer 2/3 neurons to the left. Animals were electroporated at E16.5; cortical slices (300 µm) were made at P28. (B) Quantification of total dendrite numbers separated into apical and basal dendrite branches. Epac2 knockdown in vivo induced a loss of basal dendrites compared to paired control cells. (C) Epac2 knockdown in vivo selectively reduced basal dendrite length when compared to paired control cells. (D) “Skeleton outline” of basal and apical arbors, separated into primary, secondary, and tertiary branches, of neurons shown in (A). (E) Quantification of basal dendrite numbers reveals that Epac2 knockdown in vivo induces a loss of higher order branches compared to paired control; blue square, control; red circles, Epac2-RNAi; black squares with error bars, mean, SEM. (F) Analysis of basal dendrite length demonstrates a selective reduction in higher order basal length. *p<0.05, **p<0.01, ***p<0.001; scale bars, 50 µm.

To eliminate the possibility of inter-individual and reporter expression variability, we took advantage of the sparse nature of IUEP gene transfer to directly compare dendritic morphology between neighboring non-Epac2-RNAi-expressing cells versus Epac2-RNAi-expressing cells within the same layer and cortical region from the same mice. Epac2-RNAi-expressing cells could be easily identified by the coexpression of pCAG-eGFP, while neighboring control cells not expressing Epac2-RNAi were identified by the lack of GFP expression. Pairs of GFP- and non-GFP-expressing neurons were filled with biocytin and stained with streptavidin-568 conjugated fluorescent probe to visualize dendritic morphology (Figure 2A). Using 2-photon laser scanning microscopy, we imaged paired layer 2/3 cells in the anterior frontal cortex, an area previously described to display abnormal circuitry in disease-related animal models [31]. Cortical slices were cut at a thickness of 300 µm to allow reconstruction of the majority of the apical and basal dendritic fields of these cells. This approach further confirmed that loss of Epac2 resulted in a specific reduction of basal dendrite complexity: Epac2-RNAi-expressing cells displayed an overall decrease in basal dendritic branch number (∼47%) and basal dendritic length (∼53%) (*p*<0.05, 0.01; Figures 2B–C, S2A–B). Furthermore, Epac2-RNAi-expressing cells had ∼30% fewer basal secondary dendrites and ∼55% fewer basal tertiary dendrites (*p*<0.05, 0.001; Figure 2D–E, Table S2). In addition, secondary basal length was reduced by ∼25% and tertiary basal length was reduced by ∼32% in Epac2-RNAi-expressing cells (*p*<0.05, 0.001; Figure 2D,F, Table S3). This effect was driven by the absence of high order branches (tertiary branches and beyond; Figure 2D–F). No effect on apical dendrite complexity (Figure S2C, Table S2) or length (Figure S2D, Table S3) was observed. Together, these data demonstrate that Epac2 signaling is required for maintaining higher order branching of basal dendrites in vivo. Interestingly, extended Epac2 knockdown in vivo also reduced dendritic spine density on both apical and basal dendrites, as compared to paired control electroporated cells (Figure S2E–G). This effect contrasted with that of acute (5-d) Epac2 knockdown in vitro [22], which did not alter dendritic spine numbers, but suggests that prolonged reductions in Epac2 signaling can have pronounced effects on basal dendrites and more subtle effects on apical dendrites.

### Knockdown of Epac2 Impairs Basal Dendrite Maintenance in Cultured Neurons

**Figure 3:**
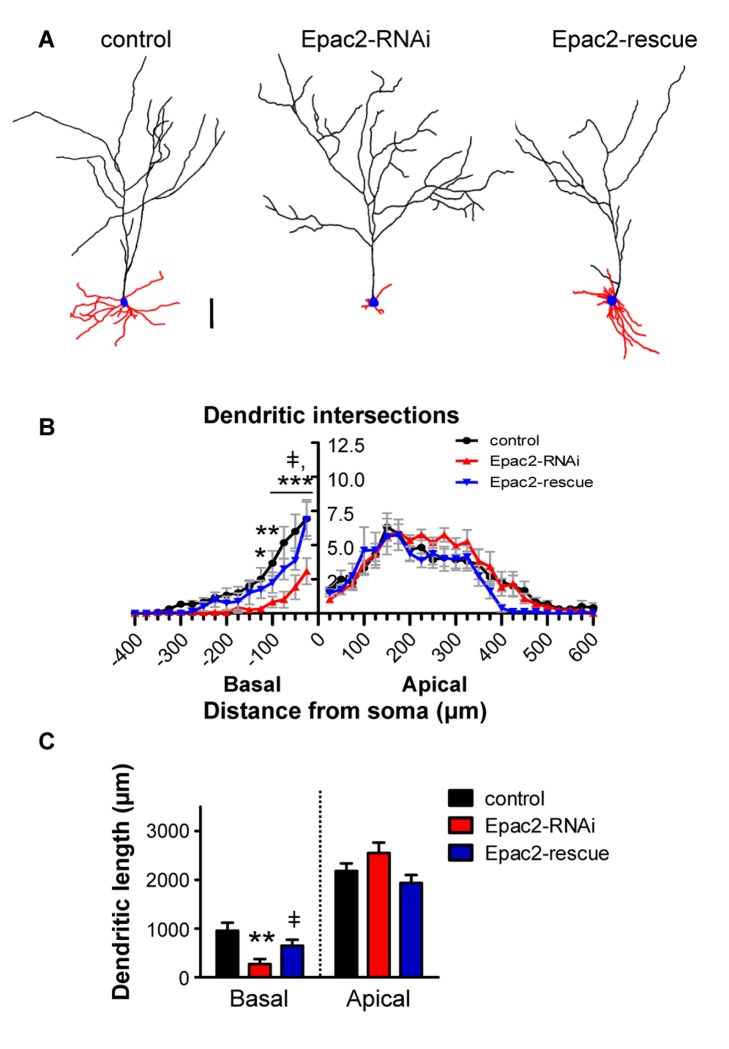
Epac2 is required for selective maintenance of basal dendrite complexity and length. (A) Binary images of cultured cortical neurons (DIV 23–28) expressing GFP, GFP+Epac2-RNAi, or GFP+Epac2-RNAi+Epac2-rescue. (B) Epac2-RNAi selectively reduces complexity of basal arbors. Expression of RNAi-insensitive Epac2-rescue recovers Epac2-RNAi-induced reduction of basal dendrite complexity to control levels. (C) Epac2-RNAi reduces basal dendritic length, while Epac2-rescue prevents this effect. ‡, difference between Epac2-rescue and Epac2-RNAi, *p*<0.05, *p<0.05, **p<0.01, ***p<0.001; scale bars, 100 µm.

Epac2 knockdown by in utero electroporation reduced Epac2 expression throughout development; however, it is not clear when Epac2 expression is required for normal dendritic morphology. To directly test the role of Epac2 in dendritic maintenance, we used an RNAi approach in mature cultured neurons, allowing perturbations in Epac2 expression levels after the dendritic arbor has already been established. This system has also been extensively used for mechanistic studies of structural plasticity [22],[32]–[34] and allows examination of the potential molecular underpinnings of dendritic architecture. A number of studies have demonstrated that mature cultured pyramidal neurons develop pyramidal morphologies with primary (classified as “apical”) and non-primary (classified as “basal”) dendrites that resemble morphologies observed in vivo (see Materials and Methods for description of criteria used for identifying apical and basal dendrites of cultured neurons) [34]–[36]. We knocked down Epac2 expression in mature (DIV 23–28) cultured cortical neurons (Figure 3A), and used Sholl analysis [37] as well as dendritic length measurements to assess the complexity and morphology of basal or apical dendritic compartments (Figure S3A–D). Consistent with our in vivo data, reduced Epac2 expression selectively decreased dendritic complexity in an asymmetric manner. Epac2 knockdown reduced basal dendritic intersections 25–175 µm from the soma, as well as basal dendrite length, without affecting apical dendrite length or complexity (basal dendritic length: length (µm), control: 956±164; Epac2-RNAi: 273±100; rescue: 647±124, *p*<0.005; Figure 3B–C). Importantly, this deficit was rescued by overexpressing an RNAi-resistant mutant of Epac2 (“Epac2-rescue”) [22]. Epac2-rescue overexpression did not significantly alter basal or apical complexity compared to control, but significantly increased basal complexity 25–100 µm from the cell body and basal dendrite length compared to Epac2-RNAi (Figure 3B–C). Similar to the effect of Epac2 knockdown in in utero electroporated neurons, Epac2 expression levels were reduced down by similar degrees (∼75%) in both apical and basal dendritic compartments in cultured cells overexpressing Epac2-RNAi (Figure S3E). Given that Epac2 knockdown occurred in mature neurons after their dendritic arbors had been established, these data suggest that Epac2 plays a role in maintenance of the basal dendritic arbor.

### Differential Regulation of Dendritic Compartments by a Disease-Associated Variant

**Figure 4:**
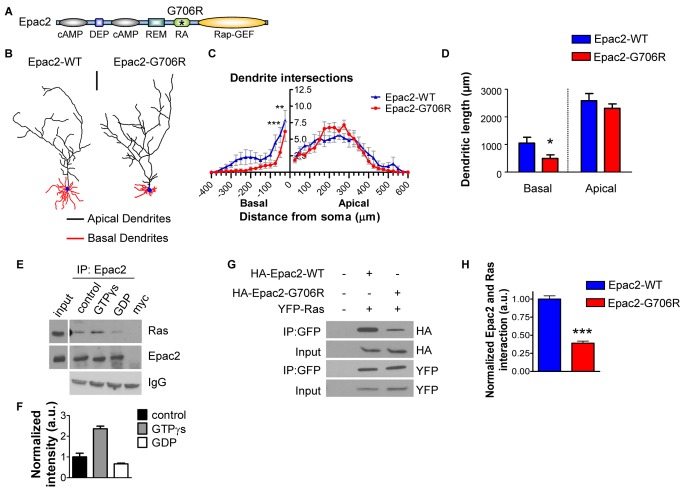
Disruption of Epac2 interaction with Ras by point mutation impairs basal dendritic maintenance. (A) Domain structure of Epac2: cAMP-binding (cAMP); domain found in Dishevelled, Egl-10, and Pleckstrin (DEP); N-terminus of Ras-exchanger motif (REM); Ras-association (RA); Rap-Guanine Exchange Factor (Rap-GEF). Asterisk indicates position of Epac2-G706R rare variant. (B) Representative binary images of cortical neurons expressing GFP+Epac2-WT or GFP+Epac2-G706R. (C) Sholl analysis of apical and basal dendrites reveals a significant decrease in basal complexity in Epac2-G706R-expressing cells. (D) Quantification of apical and basal dendrite length. (E) Ras activation by GTPγs results in stronger coimmunoprecipitation of Epac2 with Ras in neurons, while inactivation of Ras by GDP reduces Ras-Epac2 interaction. (F) Quantification of (E). (G) Epac2-G706R point mutation exhibits impaired interaction with Ras in hEK293 cells. (H) Quantification of G. *p<0.05, **p<0.01, ***p<0.001; scale bar, 100 µm.

Rare protein-coding variants of the EPAC2 gene have previously been identified in several subjects with autism [29]. One of these missense mutations (Epac2-G706R), detected in four human subjects with autism from two families, is located within the Ras association (RA) domain of Epac2 (Figure 4A), suggesting that it may affect one of Epac2's functional domains. To investigate the effect of this mutation on neuronal morphology, we expressed either Epac2-G706R or its wildtype counterpart in cultured cortical neurons. Expression of Epac2-G706R (Figure 4B), followed by Sholl analysis, revealed a robust selective decrease in basal dendrite complexity 50–100 µm from the soma, compared to Epac2-WT, with no effect on apical dendrites (Figure 4C). Furthermore, overexpression of Epac2-G706R reduced basal dendrite length relative to Epac2-WT (*p*<0.05; Figure 4D), but did not affect apical dendritic length (Figure 4D). We have previously shown that overexpression of Epac2-G706R in neurons does not affect basal Rap-GEF activity or dendritic spine morphology [22]. Comparison of the effects of Epac2-G706R overexpression to GFP alone revealed a decrease in basal complexity 25–50 µm from the soma, but no change in apical complexity, basal length, or apical length (Figure S4A–C), suggesting that Epac2-G706R is a loss-of-function mutation. Taken together, these data suggest that Epac2-G706R, a variant that occurs in human patients, specifically alters basal dendrite maintenance, without affecting apical dendritic structure, synaptic morphology, or baseline Rap activation levels.

We next reasoned that the location of the single amino-acid mutation in the Epac2 protein might offer insight into the mechanisms of asymmetric maintenance of dendritic compartments. The small GTPase Ras and its signaling partners have been implicated in neuronal morphogenesis [20],[38]–[42]. Given that the G706R mutation is within Epac2's Ras-association (RA) domain (Figure 4A), we hypothesized that this mutation might alter Epac2's interaction with Ras and that abnormal association with Ras could underlie the dendritic effects induced by Epac2-G706R. Epac2 has been shown to interact with Ras in non-neuronal cells [43], but this interaction has not yet been established in cortical neurons. We therefore tested whether Epac2 interacted with Ras in rat cortical neurons by coimmunoprecipitation. We found that Ras coimmunoprecipitated with Epac2 in mature cortical neurons (DIV 25) (Figure 4E). This interaction was dependent on the activation state of Ras: Ras activation by incubation with GTPγS enhanced the interaction between Ras and Epac2, whereas Ras inhibition by treatment with GDP reduced the interaction (*p*<0.05, 0.001; Figure 4F). This interaction was further confirmed by ectopic expression of HA-tagged Epac2-WT alone or with YFP-Ras in hEK293 cells (Figure S4D). We then tested the ability of Epac2-G706R to interact with Ras by coexpressing YFP-Ras with HA-Epac2-WT or HA-Epac2-G706R in hEK293 cells, and immunoprecipitating with YFP-Ras (Figure 4G). Indeed, quantitative analysis of coimmunoprecipitation revealed that Epac2-G706R displayed significantly impaired Ras interaction (*p*<0.001; Figure 4H). These results demonstrate that a naturally occurring Epac2 variant specifically alters basal dendritic architecture, and that interaction with Ras may be a key feature of Epac2's role in regulating basal dendritic maintenance.

### Ras Signaling Controls Basal Dendrite Complexity

The findings that Epac2 is required for the maintenance of basal (non-primary) dendrites, and that a rare coding variant that specifically disrupts Epac2's interaction with Ras mimics this selective morphological phenotype but has no affect on Epac2's basal Rap-GEF function [22], suggest a role for Epac2 and Ras signaling in the maintenance of basal dendrite complexity. Ras is a small GTPase that has been strongly linked to structural plasticity in neurons [20],[39]–[42],[44],[45], but its specific role in the maintenance of basal dendrite complexity in mature neurons has not been directly tested.

**Figure 5:**
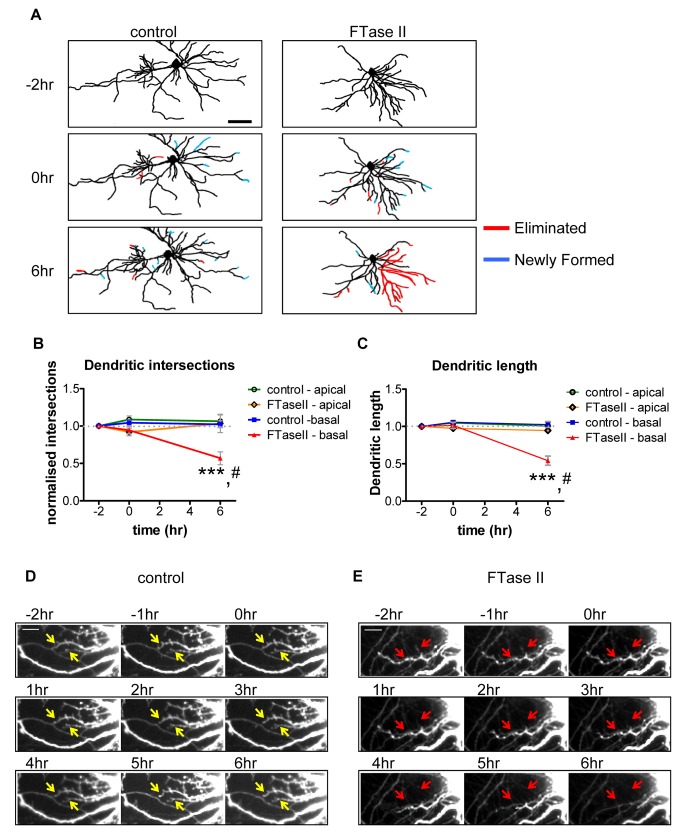
Ras inhibition induces basal dendrite retraction. (A) Binary image of basal dendrites of live-imaged cells at time points −2, 0, and 6 h of FTaseII (200 nM) or vehicle treatment. Cyan lines indicate newly formed dendrites; red lines indicate eliminated dendrites. Under control conditions, loss and gain of dendrites remains constant over imaging period. Treatment with FTaseII results in a dramatic loss of basal dendrites after 6 h of treatment. (B–C) Quantification of apical and basal arbor complexity (B) and apical and basal dendritic length (C). Data are normalized to −2 h time point. *, difference between control and FTaseII condition; #, difference between time points. (D–E) Frame-by-frame montage of basal dendrite stability under control (vehicle-treated) conditions (D; white arrows indicate stable dendrites), or of basal dendrite retraction following FTaseII treatment (E; red arrows indicate retracting basal dendrite). #, ***p<0.001; scale bars, 100 µm (A), 10 µm (D, E).

Thus, we tested whether disruption of endogenous Ras activity by the farnesyl transferase inhibitor II (FTaseII) could affect either apical or basal dendrite maintenance. We used time-lapse imaging of live mature (DIV 25) cultured cortical pyramidal neurons, expressing GFP and treated with either vehicle or FTaseII (200 nM) and measured dendritic complexity and length before and after treatment (Figure 5A and S5A). Imaging of neurons for 2 h prior to treatment revealed a remarkable stability of the dendritic arbor (Figure 5A–E) with an almost equal gain and loss of apical and basal dendrites. Imaging of apical and basal dendrites for 6 h following vehicle treatment did not reveal any changes in basal or apical dendrite complexity or length (Figure 5A–E). In contrast, incubation with FTaseII resulted in a robust retraction of basal dendrites over 6 h, as demonstrated by a reduction of basal complexity (normalized basal dendrite intersections: 0 h: 1.04±0.03 versus 0.95±0.05; 6 h 1.02±0.5 versus 0.56±0.08; control versus FTaseII, *p*<0.001; Figure 5A–B), and a loss of dendritic length (normalized basal dendrite length: 0 h: 1.06±0.02 versus 1.01±0.04; 6 h: 1.02±0.4 versus 0.54±0.06; control versus FTaseII, *p*<0.001; Figure 5A,C). This loss of length, driven by a progressive retraction of basal dendrites, was not seen in vehicle-treated neurons (Figure 5D–E). There was no change in apical dendrites (Figure 5B–C).

We further confirmed these results in neurons fixed following treatment with FTaseII or vehicle for 6 h (Figure S5B–D). Sholl analysis revealed that FTaseII treatment specifically reduced basal complexity 25–100 µm from the soma (Figure S5C) and basal dendritic length (basal dendrite length (µm); control, 1,405±221; FTaseII, 602±155, *p*<0.05; Figure S3D) with no effect on apical dendrite complexity or length (Figure S5C–D). The very specific effect of short-term FTaseII treatment on the dendritic tree is quantitatively similar to that of Epac2 knockdown or Epac2-G706R overexpression (which displays impaired Ras binding), suggesting that interference with Ras signaling, but not other potential targets of FTaseII, in cortical neurons results in a selective reduction of basal complexity.

To further investigate the impact of interference with Ras signaling on dendritic architecture, we mimicked Ras inhibition by expression of a dominant-negative Ras mutant (Ras S17N; RasDN). Expression of RasDN alone (Figure S5E–F), followed by Sholl analysis, revealed a robust selective decrease in basal dendrite complexity 50–100 µm from the soma, compared to GFP (control), with no effect on apical arbors (Figure S5F), paralleling the effects seen following short-term FTaseII treatment. Furthermore, overexpression of RasDN reduced basal dendrite length relative to control (basal dendrite length (µm); GFP, 1,704±122; RasDN, 987±198, RasDN+Epac2-WT 1,519±160, *p*<0.05; Figure S5E–F), but did not affect apical dendritic length (Figure S5G). Importantly, co-expression of Epac2-WT was sufficient to rescue RasDN-induced loss of basal dendrites and basal dendritic length (Figure S5E–G). These data provide further support for the role of the Ras/Epac2 pathway in the maintenance of basal, but not apical, dendrites.

### Asymmetric Distribution of the Ras/Epac2/Rap Pathway Across the Dendritic Arbor

**Figure 6:**
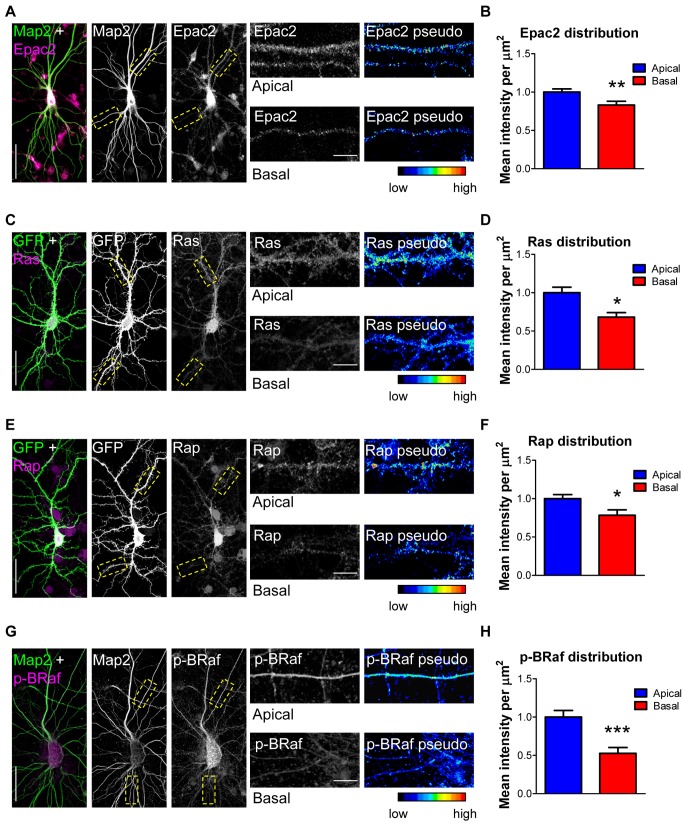
Asymmetric distribution of Ras/Epac2/Rap/p-BRaf in cortical neuron dendrites. (A) Distribution of Epac2 in apical and basal dendrites. Endogenous MAP2 indicates dendrites. (B) Epac2 fluorescence intensity (normalized to unit area, µm^2^, and indicated by pseudocoloring) is reduced in secondary basal dendrites. (C–F) Analysis of Ras (C–D) or Rap (E–F) distribution revealed that Ras and Rap are asymmetrically distributed over apical and basal dendrites. GFP outlines dendrite morphology. (G) Distribution of phosphorylated (active) BRaf in apical and basal dendrites. Endogenous MAP2 indicates dendrites. (H) p-BRaf levels are reduced in basal dendrites compared to apical dendrites. *p<0.05, **p<0.01, ***p<0.001; Scale bars, 35 µm (A, C, E, G), 5 µm (A, C, E, G insets).

The distribution of the Ras, Epac2, and Rap proteins across the dendritic tree of cortical neurons has not yet been examined. We thus compared the relative amounts of Epac2, Ras, and Rap immunostaining intensity in basal versus apical dendrites of cortical neurons in culture. All intensity measurements were limited to secondary apical or basal dendrites and were normalized to unit area (µm^2^) to ensure that measurements of protein content between apical and basal dendrites of different thicknesses were comparable (Figure 6). We observed more intense labeling for each protein in apical dendrites than in basal dendrites (*p*<0.05, 0.01; Figure 6A–F). We also examined the distribution of phosphorylated (active) BRaf (p-BRaf), a direct target of both Ras and Rap small GTPases [20],[22]. As with Ras, Epac2, and Rap, we found that p-BRaf was more abundant in apical dendrites than in basal dendrites (*p*<0.001; Figure 6G–H). Interestingly the same subcellular distribution was observed for overexpressed Epac2-WT and Epac2-G706R (*p*<0.01; Figure S6A–B). Epac2-G706R signal was reduced compared to that of Epac2-WT in basal dendrites, but not apical dendrites, in these cells (*p*<0.05; Figure S6C). In order to determine whether this asymmetric pattern was specific for Ras/Epac2/Rap pathway, we also examined the distribution of kalirin-7, a GEF for the small GTPase Rac, and the phosphorylated (active) form of p21-activated kinase (p-PAK), a direct downstream effector of Rac [46]. In contrast with the above findings, kalirin-7 and p-PAK immunofluorescence was equally distributed across apical and basal arbors (Figure S6D–E), indicating that the asymmetric distribution of Ras/Epac2/Rap was specific to this pathway.

**Figure 7:**
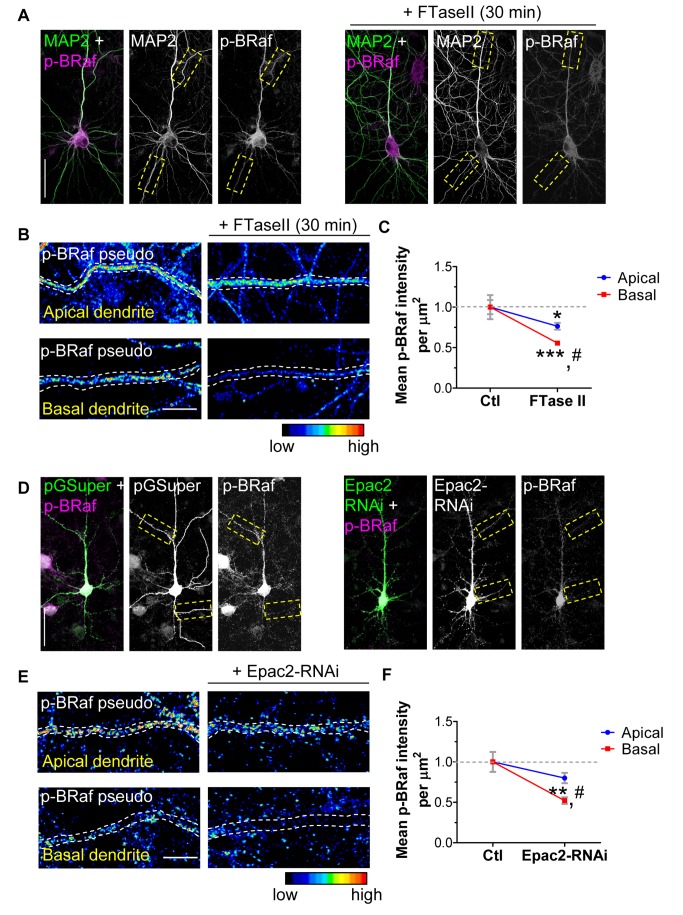
Asymmetric regulation of BRaf phosphorylation in cortical neuron dendrites. (A–C) Inhibition of Ras signaling by FTaseII (30 min, 200 nM) reduced p-BRaf immunofluorescence in basal dendrites compared to apical dendrites. Yellow boxes in (A) indicate areas in high magnification in (B). Endogenous MAP2 indicated dendrites. p-BRaf fluorescence intensity was normalized to unit area (µm^2^) and is indicated by pseudocoloring. White dashed lines outline dendrite. (C) Quantification of p-BRaf immunofluorescence in basal versus apical dendrites after 30 min FTaseII treatment relative to control apical levels (dashed gray line). (D–F) Inhibition of Epac2 function by Epac2-RNAi resulted in selective and robust reduction of p-BRaf immunofluorescence in basal dendrites compared to apical dendrites. Yellow dashed boxes in (D) indicate areas in high magnification in (E). Overexpressed GFP outlined dendrite morphology. p-BRaf fluorescence intensity was normalized to unit area (µm^2^) and is indicated by pseudocoloring. White dashed lines outline dendrite. (F) Quantification of p-BRaf immunofluorescence in basal versus apical dendrites in Epac2-RNAi-expressing cells relative to control apical levels (dashed gray line). **p<0.01, ***p<0.001 compared to control levels; #*p*<0.001 compared to apical p-BRaf levels after treatment (C, F); scale bars, 35 µm (A, D), 5 µm (B, E).

Because we observed asymmetry in the levels of Epac2/Ras/Rap across dendritic compartments of cortical neurons, we wondered if perturbations of this pathway might lead to differential signaling output in basal versus apical dendrites. To address this question, we specifically inhibited Ras signaling or reduced Epac2 expression by RNAi throughout the neurons, and examined p-BRaf immunofluorescence in individual dendritic compartments. Inhibition of Ras using FTaseII (Figures 7A, S7A) resulted in a ∼22% reduction in p-BRaf levels in apical dendrites compared to control (Figures 7A–C, S7A), but produced a more profound reduction in p-BRaf levels in basal dendrites (∼45% compared to control basal levels), which was significantly different to both control and FTaseII-induced apical p-BRaf levels (mean p-BRaf intensities relative to control levels: FTaseII; apical 0.76±0.04; basal 0.55±0.01; *p*<0.001; Figures 7B–C, S7B–C). Examination of endogenous Epac2 levels in control (pGSuper) and Epac2-RNAi cells revealed that Epac2 expression was less abundant in basal dendrites than apical dendrites after Epac2 knockdown (reduced by ∼37% compared to apical levels; Figure S7D), suggesting that asymmetric localization of Epac2 is maintained under knockdown conditions. Moreover, comparison of Epac2 immunofluorescence in apical or basal compartments in control versus Epac2-RNAi-expressing neurons, relative to Epac2 levels in control apical dendrites, revealed that Epac2 expression in apical dendrites of Epac2-RNAi cells was not significantly different from Epac2 levels in control basal dendrites (Figure S7E), suggesting that apical dendrites may contain enough Epac2 even in the knockdown condition to preserve apical dendrite morphology. Epac2 knockdown (Figures 7D, S7F) resulted in a greater reduction of p-BRaf levels in basal dendrites (∼47%) versus apical dendrites (∼20%), when compared to control levels (mean p-BRaf intensities relative to control levels: Epac2-RNAi; apical 0.80±0.07; basal 0.52±0.04; *p*<0.001; Figures 7D–F, S7F–H). Collectively, these data suggest that the signaling output of the Ras/Epac2/Rap pathway is asymmetric across dendritic compartments of cortical neurons.

## Discussion

Here we showed that Epac2 is important for the selective maintenance of basal dendrite complexity in cortical neurons. Utilizing a rare coding variant of Epac2, found in human patients, to probe the molecular and cellular functions of Epac2 in the context of dendritic complexity of cortical pyramidal neurons, we identified Ras as a signaling partner of Epac2 in this pathway. Our findings support a model in which Epac2, as a Rap-GEF, enables crosstalk between two morphogenic GTPase signaling pathways to maintain basal dendrites.

The importance of maintaining dendritic architecture is illustrated by observations of dendritic complexity in pathological analysis of patients with neuropsychiatric and neurodevelopmental disorders. Reduced dendritic arborization occurs in patients with psychiatric disorders with delayed onset, including schizophrenia [3], autism spectrum disorders [4],[5], and disorders comorbid with autism, such as Rett [6] and Down syndromes [7]–[9]. In Down syndrome, loss of dendritic arbors occurs in a progressive manner: prior to 2 y of age, dendritic hypertrophy was observed in the cortex of subjects with Down syndrome; thereafter, dendritic arbors were reduced in complexity relative to controls [7]–[9], suggesting an initial period of dendritic overgrowth followed by a later stage in which maintenance mechanisms are potentially disrupted or lost. An early study of visual cortex from subjects with Down syndrome revealed a selective loss of basal dendrite complexity [47], though subsequent studies have also detected apical deficits [8]. Rett syndrome, a monogenic disorder frequently accompanied by an autistic phenotype, features dendritic deficits, usually in basal dendrites and occasionally in apical dendrites [6]. Individuals with autism exhibited reduced dendrite numbers and dendritic cytochemical markers in the cortex [4] and hippocampus [5]. Thus, a more complete understanding of molecular mechanisms that contribute to the maintenance of specific aspects of the dendritic arbor may hasten the development of therapeutic strategies that aim to prevent the apparent progressive loss of dendritic complexity and to preserve functional cortical circuits in patients with neurodevelopmental and neuropsychiatric disorders in which dendritic structure is affected.

Several lines of evidence support the selective regulation of apical versus basal dendritic compartments. Environmental enrichment has been shown to selectively increase basal dendrite length, while stress can reduce basal dendritic length, in layer 2/3 cells of the auditory cortex [15]. Sensory deprivation by whisker trimming during a critical developmental window, between P9 and P15 in rats, delayed normal basal dendritic elaboration of layer 2/3 pyramidal cells in the barrel cortex [17]. PTEN knockout results in selective outgrowth of apical dendrites, demonstrating that inhibition of mTOR blocks continued apical but not basal dendrite growth under stable conditions in mature animals [18]. Particularly relevant to Epac2 signaling is the recent report that knockout of dopamine receptor D1 in mouse cortex results in selective basal dendrite loss [19], as Epac2 is also regulated by D1/cAMP signaling [22]. Given the ability of Epac2 to selectively regulate the basal arbor, signaling through Epac2 may be a key mechanism for control of select dendritic compartments. A current hypothesis is that the establishment and elaboration of apical and basal arbors occur at distinct time points, with basal dendrites developing after apical dendrites, and thus may involve distinct regulatory mechanisms [48]. Our knockdown data indicate that Epac2 is required for the maintenance of higher order basal dendrite branches. Elaboration of higher order basal dendrite branching, which increases basal dendrite complexity, occurs subsequent to the formation of the primary proximal basal arbor and the apical arbor [48]. Interestingly, this time point (3 wk postnatal) coincides with a dramatic increase in Epac2 expression in cortical neurons [22]. Thus, our findings support a role for Epac2 in regulating the maintenance of basal dendritic complexity once these complex arbors have been initially established.

Here we were guided by a rare coding mutation, naturally occurring in human subjects with autism, to identify the Ras/Epac2 interaction as important for the control of basal dendrite complexity in cortical neurons. Interestingly, a different point mutation in the RA domain of Epac2, identified through sequence analysis rather than occurrence as a rare variant in human patients, has also been shown to disrupt the interaction with Ras in COS cells [49], suggesting that single-residue sites in this domain of Epac2 may be crucial for its function in response to Ras, and that even single amino acid mutations occurring as rare variants may have functional or pathological consequences. Our strategy exemplifies how mutations identified in humans with neurodevelopmental or psychiatric disorders, beyond their relevance for disease, could provide functional insight into novel mechanisms underlying brain development and connectivity. A growing number of rare single amino acid mutations have been identified in neuropsychiatric disorders by recent genetic studies, and with the advent of whole exome or genome sequencing, their numbers are expected to increase dramatically [50]. While their significance for disease etiology remains to be elucidated, our approach taken in this study shows that such mutations might help identify cellular mechanisms that control crucial cellular processes, including dendrite arborization. About 20% of single amino acid mutations are thought to be damaging, with another 53% being mildly deleterious [51]. Given that rare mutations are thought to make up a significant fraction of the genetic architecture of complex diseases, functional characterization of such mutations may provide novel insight into both physiology and pathophysiology.

In this study, we show that a pathway involving Ras/Epac2/Rap contributes to the maintenance of basal dendrite complexity. The G706R point mutation disrupted the Ras-Epac2 interaction and reduced basal complexity, and Ras inhibition experiments using FTaseII or RasDN overexpression specifically affected basal dendrite maintenance, suggesting that this pathway exerts specific control over basal dendritic complexity in pyramidal neurons. Our results establishing a role for Epac2 in linking Ras and Rap signaling to dendrite maintenance in mature cortical neurons are consistent with a number of previous findings. Dominant-negative Rap1-expressing layer 5 pyramidal neurons exhibit deficits in basal dendrite arborization during development [38]. In non-neuronal cells, activated Ras has been shown to recruit Epac2 to the plasma membrane, thereby activating membrane-associated pools of Rap [43]. In adrenally derived PC12 cells, Ras activation can recruit Epac2 to the plasma membrane, activate membrane-associated pools of Rap1, and induce the outgrowth of neurite-like structures [49]. Recent characterizations of EPAC null mice reveal cognitive and behavioral phenotypes, illustrating the importance of Epac in complex behavior and brain function [25]; however, dendritic architecture of cortical neurons was not measured in this mouse model. Our data implicating Epac2 in the maintenance of basal arbors of cortical neurons provide a potential mechanism for the disruption of neuronal circuitry upon perturbations of this pathway. Our observation of asymmetric distribution of Epac2, Ras, and Rap proteins is consistent with the selective effect of reduced Ras/Epac2 signaling on the maintenance of basal dendrites. Indeed, it is reasonable to expect that other redundant mechanisms are employed for the active maintenance of apical dendritic architecture, which may require more stability during the life of the neuron. Our data showing that disruption of a single pathway can alter basal maintenance are consistent with the intrinsic dynamism of basal dendrites, due to the higher demands for plasticity driven by sensory and inhibitory inputs to this compartment [11],[13],[14]. Taken together, our data support a model in which Epac2 couples with Ras signaling and actively maintains basal dendrites in cortical pyramidal neurons.

## Materials and Methods

### Reagents

Farnesyl transferase inhibitor II was purchased from EMD Biosciences. We purchased the following antibodies: mouse anti-GFP monoclonal (Millipore), chicken anti-GFP polyclonal (Abcam), rabbit anti-Epac2 polyclonal and mouse anti-Epac2 monoclonal (Santa Cruz), rabbit anti-HA polyclonal (Enzo) and mouse anti-HA monoclonal (Santa Cruz), mouse anti-Ras monoclonal (Upstate), mouse anti-Myc monoclonal (Developmental Studies Hybridoma Bank, Iowa), mouse anti-MAP2 monoclonal (Millipore), and rabbit anti-Rap polyclonal antibody (Millipore). A rabbit anti-GFP polyclonal antibody was a gift from Dr. Richard Huganir (Johns Hopkins University). The pCAG-EGFP construct was a kind gift from Atsushi Kamiya, Johns Hopkins University. The pEGFP-N2 construct was obtained from Clontech. Constructs encoding shRNA specific for Epac2 and a rescue construct (an HA-Epac2 construct containing three silent point mutations in the RNAi target sequence) were previously generated and validated [22].

### Neuronal Cultures and Treatments

Dissociated cultures of primary cortical neurons were prepared from E18 Sprague-Dawley rat embryos as previously described [22]. On DIV 21–23, neurons were transiently transfected for 4 h with plasmids (1–3 µg DNA) using Lipofectamine 2000 (Invitrogen). For experiments utilizing soluble GFP, cultures were allowed to express the transfected constructs for 2 d. For RNAi and Epac2 mutant, constructs were expressed for 5 d. Rats were used in accordance with ACUC institutional and national guidelines under approved protocols. Treatment of live cultured neurons was performed in ACSF essentially as previously described [22]. Briefly, cultured neurons were transfected with GFP, and allowed to express for 2 d. Neurons were then pre-incubated in ACSF for 1 h, imaged 2 h and 1 h before beginning of treatment, and then imaged every hour for 6 h after beginning of treatment with either FTaseII (200 nM) or vehicle. Coverslips were kept in culture plates throughout the experiment, and were returned to a 37°C incubator between imaging timepoints. Micrographs of healthy GFP-expressing neurons with pyramidal morphologies were acquired using a 10× objective (NA = 0.17) and a Zeiss AxioCam MRm CCD camera. Dendrites were traced and binarized in ImageJ as described below. It is of note that whereas farnesyl transferase inhibitors were initially developed for their ability to inhibit Ras activity, a number of other proteins are also farnesylated, and therefore the potential contribution of other proteins to this specific loss of basal dendrites cannot be excluded.

### Visualization and Quantification of Dendritic Spine Morphology in In Utero Electroporated Sections

For dendritic spine morphologies in vivo (Figure S2), images of dendritic spines on biocytin-filled neurons were acquired with a Zeiss LD Lci Plan Apochromat 25×/0.8NA multi-immersion lens (440842-9870-000000) with a digital zoom of 4. Volume imaging was acquired with 15–35 optical sections taken in 0.75 µm focal steps (2.13 µm axial resolution). For each condition, 5 neurons were imaged. Two dendrites between 50 and 100 µm in length per cell were measured: only spines on tertiary apical or secondary basal dendrites were imaged to reduce variability. Dendritic spine density (number of spines per 10 µm) was calculated using ImageJ.

### Quantitative Immunofluorescence

Cultured pyramidal neurons were fixed, immunostained, and imaged as previously described [22]. Protein clustering was imaged as z-series taken at 0.37 µm intervals using a Zeiss LSM5 Pascal confocal microscope and a 63× objective (NA = 1.4). Two-dimensional maximum projection images were reconstructed and analyzed using MetaMorph software (Molecular Devices, Sunnyvale, CA, USA). Images were background-subtracted and thresholded equally to include clusters with intensity at least 2-fold above the adjacent dendrite. Regions along dendrites were manually outlined, and the linear density (number per 100 µm of dendrite length) and total gray value (total immunofluorescence intensity) of each cluster was measured automatically. Cultures that were directly compared were stained simultaneously and imaged with the same acquisition parameters. Experiments were carried out blind to condition and on sister cultures.

### Coimmunoprecipitations

Coimmunoprecipitations (coIPs) from hEK293 cells or rat cortical tissue were performed as previously described [22], using RIPA buffer (in mM: 150 NaCl, 10 Tris-HCl, pH 7.2, 5 EDTA, 0.1% SDS, 1% Triton X-100, 1% Deoxycholate, plus inhibitors). Precleared lysates were incubated with 2.5–5 µL of antibody for 3 h; 60 µL of protein-A Sepharose was added for 2 h at 4°C, after which samples were washed 3 times with 0.5 ml RIPA buffer, boiled for 5 min at 95°C in Laemmli buffer, and analyzed by SDS-PAGE and Western blotting. For treatment with GTPγS or GDP, cortical neurons were lysed in Mg^2+^ lysis buffer containing protease inhibitors. Cell lysates were then incubated with 100 µM GTPγS or 1 mM GDP at 30°C for 30 min. Reaction was stopped by the addition of 60 mM MgCl_2_. Cell lysates were harvested as described above.

### In Utero Electroporation (IUEP)

C57BL6 female mice were checked for vaginal plugs (E0), and electroporation was performed at E16.5. After proper sedation, both uterine horns were removed and placed on sterile, warm, and PBS-wetted pads. DNA solution was loaded into beveled glass micropipettes (100 µm oblique opening), and 0.26 µl was injected into the lateral ventricle through the uterus wall (4 injections; 65 nL/injection) using a nanojector (Drummond Nanoject II). DNA was electroporated into the neural precursor populations that reside on the ventricular zone by directed electroporation, by placing the (+) end of the electrode toward the developing neocortex. Unipolar electric pulse of 40 V was generated (BTX ECM830), and a total of five 50 ms pulses at an interval of 100 ms were applied to the cerebral wall. After electroporation, embryos were placed back into the abdominal cavity, and the rectus abdominis and abdominal oblique muscles were sutured with 5-0 coated vicryl suture for quick absorption and fast recovery. The skin was closed with LiquiVet tissue adhesive. Mice were allowed to recover and give natural birth. Injected animals were collected at P28 for further investigations. A preparation of DNA 3∶1 molar ratio was used to mix pCAG-EGFP construct [52], which expresses EGFP under the chicken beta actin promoter, and Epac2-RNAi cloned into pGSuper expression vector [22]. 0.05% FastBlue was added to visualize DNA. In control experiments, pGSuper was mixed with pCAG-eGFP construct.

### Imaging of Dendrites in 50 µm IUEP Slices

Electroporated mice were anesthetized with sodium pentobarbital at 50 mg per gram of body weight, and fixed by transcardial perfusion of 4% paraformaldehyde in 0.1 M sodium phosphate buffer (pH 7.4) at P28. Brains were dissected out and sectioned into 50 µm coronal sections and immunostained with a chicken anti-GFP polyclonal antibody (Abcam) as floating sections, before being mounted onto glass slides and covered with glass coverslips. Cells exhibiting intact and healthy secondary and tertiary apical and basal dendritic arbors were imaged by taking 1 µm serial optical sections, 35–45 optical sections per cell, using a Zeiss LSM5 Pascal confocal microscope and a 40× objective (NA = 1.3). Following acquisition, images were projected as 2-D Z-projections using Fiji6/ImageJ (http://imagej.nih.gov/ij/; NIH, Bethesda, MD, USA). Dendrites were analyzed using NeuronJ plugin [53] for Fiji6/ImageJ. Between 12 and 15 cells per condition were analyzed.

### Biocytin Filling of Paired Cells

At P28, electroporated mice were deeply anesthetized with isoflurane and their brains were quickly removed. Brain sections were cut at a thickness of 300 µm using an off-sagittal slice angle to preserve apical and basal tufts of layer 2/3 cortical neurons of the anterior frontal cortex [31]; sections were cut in ice-cold carbogenated choline solution (in mM: 110 choline chloride, 25 NaHCO_3_, 2.5 KCl, 1.25 NaH_2_PO_4_, and 0.5 CaCl_2_, 7 MgSO_4_, 25 D-glucose, 11.6 sodium ascorbate, 3.1 sodium pyruvate). Slices were transferred to carbogenated artificial cerebrospinal fluid (ACSF, in mM: 126 NaCl, 2.5 KCl, 26 NaHCO_3_, 2 CaCl_2_, 1 MgCl_2_, 1.25 NaH_2_PO_4_, and 10 D-glucose) and incubated for 30 min at 35°C. They were then maintained at room temperature for the remainder of the intracellular labeling procedure. To maximize the amount of dendritic arbor, we selected neighboring pairs of GFP-positive, Epac2-RNAi-expressing, and non-fluorescent control neurons with somata deeper than 60 µm from the surface of the brain slice (average depth = 89±3.9 µm). Using a micropipette filled with biocytin intracellular solution (in mM: 10 biocytin, 126 K-methylsulfate, 4 KCl, 10 HEPES, 4 ATP, 0.3 GTP, and 10 phosphocreatine), we dialyzed the neurons for at least 15 min and then allowed the cells to recover for at least 30 min before fixing the slices in 4% paraformaldehyde in 0.1 M sodium phosphate buffer (pH 7.4). Slices were then immunostained with a fluorescent streptavidin-568 conjugate (Invitrogen) and chicken anti-GFP polyclonal antibody (Abcam) as floating sections. Sections were mounted under a #1.5 coverslip with 2 #1 coverslips (∼150 µm thickness) placed either side of the section to avoid damage to the tissue.

### 2-Photon Laser Scanning Microscopy (2PLSM)

Images were taken with a Prairie Ultima 2-photon in vivo microscope, using a Mira 900F laser at a wavelength of 795 nm (6 nm bandwidth) to locally excite both Alexa-488 and -568 nm fluorescence, with a Zeiss LD Lci Plan Apochromat 25×/0.8NA multi-immersion lens (440842-9870-000000). Volume imaging was acquired with 300–375 optical sections taken in 0.75 µm focal steps (2.13 µm axial resolution). The objective lens lateral resolution was defined to be 0.43 µm with 795 nm and NA = 0.8 and captured with pixels of 0.22 µm (2,048×2,048, 440 µm field of view), 4 µs pixel dwell time. Best performance was achieved with Cargill Type FF immersion oil, an index of 1.479, and using the glycerol with cover slip objective lens correction collar setting. Only pairs of cells exhibiting intact healthy secondary and tertiary apical and basal dendrites were imaged and used for quantification. Following acquisition, images were projected as 2-D Z-projections using Fiji6. Dendrites were analyzed using NeuronJ plugin for Fiji6/ImageJ. 5 animals were analyzed.

### Dendrite Visualization and Quantitative Morphometric Analysis

To quantify dendritic morphology in vitro, cultured neurons expressing GFP were imaged using a 10× objective (NA = 0.17), and micrographs were acquired using a Zeiss AxioCam MRm CCD camera. Dendrites were traced and binarized in ImageJ. The axon was identified by its distinct morphology and was eliminated from quantification. The following criteria for identifying apical and basal dendrites in cultured neurons were used. “Apical” dendrites were defined as the longest single protrusion, also referred to as the primary dendrite, which has the largest diameter proximal to the cell body [35],[36], whereas “basal” dendrites were identified as smaller and shorter protrusions, with a smaller diameter proximal to the cell body, compared to the primary dendrite (Figure S3A–B). Examination of Golgi outposts in vitro and in vivo has demonstrated that the longest dendritic protrusions (primary dendrite) contain Golgi complexes in cultured neurons, and that in vivo, Golgi complexes are found in apical dendrites [34],[35]. Indeed, we found that in the majority (>90%) of neurons in our cultures, only one dendrite, typically the longest one, was positive for giantin, a marker for the Golgi complex (Figure S3B), which we have classified as the apical dendrite [34]. Dendritic length was measured in MetaMorph. For Sholl analysis, we used the Sholl analysis plugin for ImageJ (http://biology.ucsd.edu/labs/ghosh/software) to measure the number of dendritic processes that intersected with concentric circles spaced 25 µm apart starting at the center of the soma. For each parameter, 7–17 cells from 3–5 experiments were measured, and images were acquired and quantified by an experimenter blind to condition.

### Statistical Analysis

For quantitative immunofluorescence experiments, coIPs, and dendrite length or number measurements, differences among condition means were identified by Student's unpaired *t* tests or ANOVAs performed in GraphPad Prism (La Jolla, CA, USA) or SPSS (Armonk, NY, USA). Tukey-b or Bonferroni post hoc analyses were used for multiple comparisons. Error bars represent standard errors of the mean. For Sholl analysis, mixed model ANOVAs (condition×distance from soma) were conducted, with distance from soma as a repeated measure. Student's paired *t* tests were used to analyze paired cell morphology.

## Supporting Information

Figure S1 In utero electroporation of Epac2-RNAi reduces basal dendrite complexity. (Related to Figure 1) (A) Schematic of in utero microinjection and electroporation of Epac2-RNAi and GFP cDNAs into embryonic mouse brain. cDNAs are microinjected into 4^th^ ventricle of E16.5 mouse embryos and electroporated into the developing mouse neocortex. Electroporated neurons migrate into cortex and establish connectivity with surrounding circuits, and morphology is imaged in slices taken at postnatal day 28. (B) Immunostaining of GFP (RNAi-positive cells), endogenous Epac2, and DAPI in 50 µm coronal sections of in utero electroporated brains (P28). Yellow arrows indicate electroporated, GFP-positive, and Epac2-RNAi (Epac2-negative) cells; white arrowheads indicate non-electroporated Epac2-positive cells. (C) Epac2 immunofluorescence intensity is decreased by ∼75% in cells expressing Epac2-RNAi compared to non-electroporated cells. ***p<0.001; scale bar, 50 µm.
(PDF)

Figure S2 Epac2 regulates higher order basal dendrite complexity, without affecting apical dendrite complexity in vivo. (Related to Figure 2) (A–B) Quantification of total apical and basal dendrite number (A) and length (B) in paired cells; blue square, control; red circles, Epac2-RNAi; black squares with error bars, mean, SEM. (C) Quantification of apical dendrite numbers, separated into primary, secondary, or tertiary and above, reveals no effect of Epac2 knockdown in vivo on apical dendrite number compared to paired control; blue square, control; red circles, Epac2-RNAi; black squares with error bars, mean, SEM. (D) Analysis of apical dendrites, separated into primary, secondary, or tertiary and above, demonstrates no effect of Epac2 knockdown on apical dendrite length. (E) Representative images of dendritic spines in tertiary apical or secondary basal dendrites in control or Epac2-RNAi-expressing layer 2/3 cortical neurons. (F) Quantification of dendritic spine number in tertiary apical or secondary basal dendrites in control or Epac2-RNAi-expressing layer 2/3 cortical neurons. Long-term expression of Epac2-RNAi significantly reduces dendritic spine number in apical and basal dendrites. (G) Epac2-RNAi reduces dendritic spine density on both apical and basal dendrites with pairs of electroporated neurons. *p<0.05, **p<0.01; scale bar, 5 µm.
(PDF)

Figure S3 Quantification of dendritic complexity and length. (Related to Figure 3) (A) Representative image of primary, classified as apical (black) and non-primary, classified as basal (red) dendrites of cortical pyramidal neurons (DIV 25). (B) Double immunostaining with MAP2 and Golgi complex marker, giantin. Primary (apical) dendrite is identified by red arrow. Note the presence of Golgi in the apical dendrite, as well as differences between apical and basal dendrite diameters. (C) Schematic of Sholl analysis of basal and apical dendrites separately. (D) Schematic of how dendrite length measurements were performed. Each dendrite was traced (red dotted line represents tracing of apical dendrites, whereas black dotted line represents tracing of basal dendrites), and total dendritic length of each compartment was calculated. (E) Quantification of Epac2 expression levels in apical or basal dendrites in either control (pGSuper) or Epac2-RNAi-expressing cells. In both compartments, Epac2 is knocked down with equal efficiency (∼75%). ***p<0.001.
(PDF)

Figure S4 Epac2-G706R overexpression impairs basal dendrite maintenance and interaction with Ras. (Related to Figure 4) (A) Representative binary traces of cortical neurons expressing GFP or GFP+Epac2-G706R. (B) Sholl analysis reveals that complexity of the basal dendrite arbor was reduced in cells expressing Epac2-G706R compared to cells expressing GFP. (C) Quantification of apical and basal dendrite length reveals no difference in dendritic length of basal arbors. (D) Epac2 and Ras coimmunoprecipitate in hEK293 cells. HA-tagged Epac2-WT and YFP-tagged Ras were expressed in hEK293 cells, and immunoprecipitated using a rabbit anti-GFP polyclonal antibody. ***p<0.001; scale bar, 100 µm.
(PDF)

Figure S5 Inhibition of Ras activity reduces basal dendritic maintenance. (Related to Figure 5) (A) Binary images of full dendritic arbors of cortical neurons shown in Figure 5A (dashed red rectangle indicates basal arbor shown in Figure 5A) at −2 hr pretreatment. Neurons were imaged live and treated with vehicle or farnesyltransferase inhibitor II (FTaseII, 200 nM) for 6 h. (B) Binary images of cortical neurons treated with FTaseII (200 nM) or vehicle for 6 h, and subsequently fixed and imaged. (C) Sholl analysis of apical and basal dendrites reveals that FTaseII treatment for 6 h selectively reduces complexity of basal arbors. (D) Quantification of mean dendritic length for apical dendrites and basal dendrites demonstrates a reduction of basal dendrite length induced by FTaseII treatment. (E) Representative binary images of cortical neurons expressing GFP, GFP+RasDN, or GFP+RasDN+Epac2-WT. (F) Sholl analysis of apical and basal dendrites reveals a significant decrease in basal complexity in RasDN-expressing cells; this effect is rescued by Epac2-WT overexpression. (G) Quantification of apical and basal dendrite length. *p<0.05, **p<0.01, ***p<0.001; scale bars, 100 µm (A, B, E).
(PDF)

Figure S6 Asymmetric distribution of ectopically expressed Epac2-WT and Epac2-G706R, but lack of asymmetric distribution of kalirin-7 and p-PAK in cortical neuron dendrites. (Related to Figure 6) (A) Distribution of HA-Epac2-WT in apical and basal dendrites. Exogenous mCherry indicates dendrites. HA-Epac2-WT fluorescence intensity (normalized to unit area, µm^2^, and indicated by pseudocoloring) is reduced in secondary basal dendrites. (B) Distribution of HA-Epac2-G706R in apical and basal dendrites. Exogenous mCherry indicates dendrites. HA-Epac2-G706R fluorescence intensity (normalized to unit area, µm^2^, and indicated by pseudocoloring) is reduced in secondary basal dendrites. (C) Comparison of Epac2 immunofluorescence levels in (A–B) normalized to Epac2-WT apical levels reveals a significant decrease in basal Epac2-G706R intensity compared to basal Epac2-WT intensity. (D) Distribution of the Rac-GEF kalirin-7 over apical and basal dendrites. Yellow dashed boxes indicate areas in high magnification to the right. Overexpressed GFP was used to outline dendrite morphology. Intensity of kalirin-7 staining was normalized to unit area (µm^2^) over a 50 µm length of secondary apical or basal dendrite to account for thickness of dendrite. Kalirin-7 fluorescence intensity is equally abundant in basal and apical dendrites. (E) Examination of p-PAK (an indicator of Rac signaling): immunostaining revealed that p-PAK fluorescence intensity is equally abundant in basal and apical dendrites. Endogenous MAP2 was used to indicate dendrites. Scale bars, 35 µm (A, B, D, E), 5 µm (A, B, D, E insets).
(PDF)

Figure S7 Asymmetric distribution and regulation of p-BRaf in cortical neuron dendrites. (Related to Figure 7) (A) Staining of endogenous p-BRaf in apical and basal secondary dendrites treated with vehicle or FTaseII. Images are high magnifications of areas in yellow dashed boxes indicated in Figure 7A. Inhibition of Ras signaling by FTaseII (30 min, 200 nM) reduced p-BRaf immunofluorescence in basal dendrites compared to apical dendrites. Endogenous MAP2 was used to indicate dendrites. (B) Quantification of p-BRaf immunofluorescence in basal versus apical dendrites following FTaseII treatment; p-BRaf fluorescence intensity was normalized to unit area, µm^2^. (C) Comparison of p-BRaf immunofluorescence in basal versus apical dendrites after 30 min FTaseII treatment relative to control apical levels (dashed gray line). (D) Quantification of Epac2 distribution in secondary apical or basal dendrites from cells expressing pGSuper (control RNAi) or Epac2-RNAi; Epac2 intensity was normalized to unit area, µm^2^. Epac2 is asymmetrically distributed with less expression in basal dendrites compared to apical dendrites in both control and Epac2-RNAi expressing cells. (E) Comparison of endogenous Epac2 fluorescence intensities in (D), normalized to control apical levels, reveals a significant decrease in Epac2 levels in control basal dendrites, but no significant difference in Epac2 levels in Epac2-RNAi apical dendrites versus control basal dendrites. (F) Staining of endogenous p-BRaf in apical and basal dendrites in the presence of pGSuper or Epac2-RNAi. Images are high magnifications of areas in yellow dashed boxes indicated in Figure 7D. Inhibition of Epac2 function by Epac2-RNAi resulted in selective and robust reduction of p-BRaf immunofluorescence in basal dendrites compared to apical dendrites. Overexpressed GFP was used to outline dendrite morphology. (G) Quantification of p-BRaf immunofluorescence in basal versus apical dendrites in Epac2-RNAi-expressing cells; p-BRaf fluorescence intensity was normalized to unit area, µm^2^. (H) Comparison of p-BRaf immunofluorescence in basal versus apical dendrites in Epac2-RNAi-expressing cells relative to control (pGSuper) apical levels (dashed gray line). *p<0.05, **p<0.01, # ***p<0.001; #, compared to control apical (C, E, H). scale bars, 5 µm (A, F).
(PDF)

Table S1 Quantification of dendritic morphology for apical and basal dendritic branch number and length in in utero electroporated 50 µm sections.
(PDF)

Table S2 Quantification of apical and basal dendritic branch number in in utero electroporated paired neurons in 300 µm sections.
(PDF)

Table S3 Quantification of apical and basal dendritic branch length in in utero electroporated paired neurons in 300 µm sections.
(PDF)

## Abbreviations

ACSF: artificial cerebrospinal fluid
DIV: days in vitro
DN: dominant negative
Epac2: exchange protein directly activated by cyclic AMP 2
FTaseII: farnesyltransferase inhibitor II
GDP: guanosine diphosphate
GEF: guanine nucleotide exchange factor
GFP: green fluorescent protein
GTPγS: guanosine 5′-O-[gamma-thio]triphosphate
HA: human influenza hemagglutinin
hEK293: human embryonic kidney 293
IUEP: in utero electroporation
p-PAK: phosphorylated p21-associated kinase
RA: Ras association
RNAi: RNA interference
WT: wild type
YFP: yellow fluorescent protein
