# Supplementary figures and images for: An Autism-Associated Variant of Epac2 Reveals a Role for Ras/Epac2 Signaling in Controlling Basal Dendrite Maintenance in Mice
Source: PLoS Biol. 2012 Jun 26;10(6):e1001350. doi: 10.1371/journal.pbio.1001350 (PMC3383751; doi:10.1371/journal.pbio.1001350)

**A**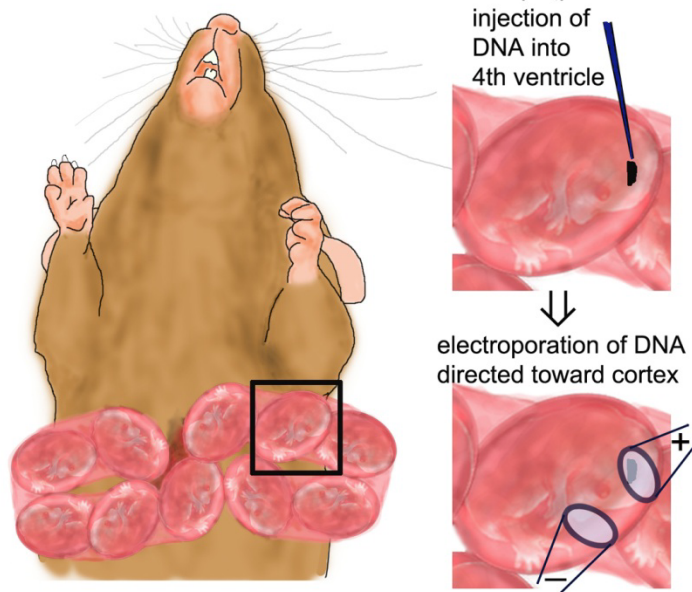**B**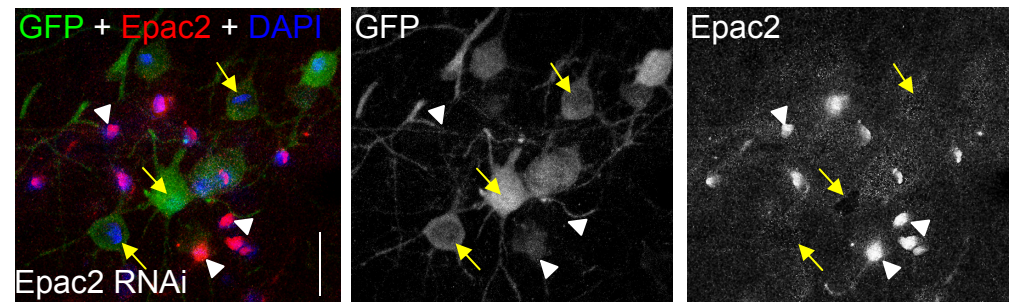**C**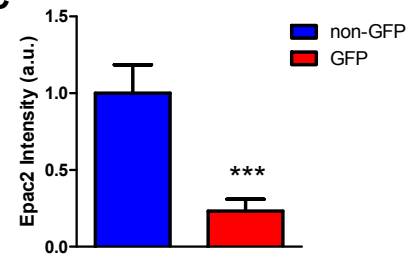

Figure S1

Supplement: Figure S1 — In utero electroporation of Epac2-RNAi reduces basal dendrite complexity. (Related to Figure 1) (A) Schematic of in utero microinjection and electroporation of Epac2-RNAi and GFP cDNAs into embryonic mouse brain. cDNAs are microinjected into 4th ventricle of E16.5 mouse embryos and electroporated into the developing mouse neocortex. Electroporated neurons migrate into cortex and establish connectivity with surrounding circuits, and morphology is imaged in slices taken at postnatal day 28. (B) Immunostaining of GFP (RNAi-positive cells), endogenous Epac2, and DAPI in 50 µm coronal sections of in utero electroporated brains (P28). Yellow arrows indicate electroporated, GFP-positive, and Epac2-RNAi (Epac2-negative) cells; white arrowheads indicate non-electroporated Epac2-positive cells. (C) Epac2 immunofluorescence intensity is decreased by ∼75% in cells expressing Epac2-RNAi compared to non-electroporated cells. ***p<0.001; scale bar, 50 µm. (PDF) [file pbio.1001350.s001.pdf]

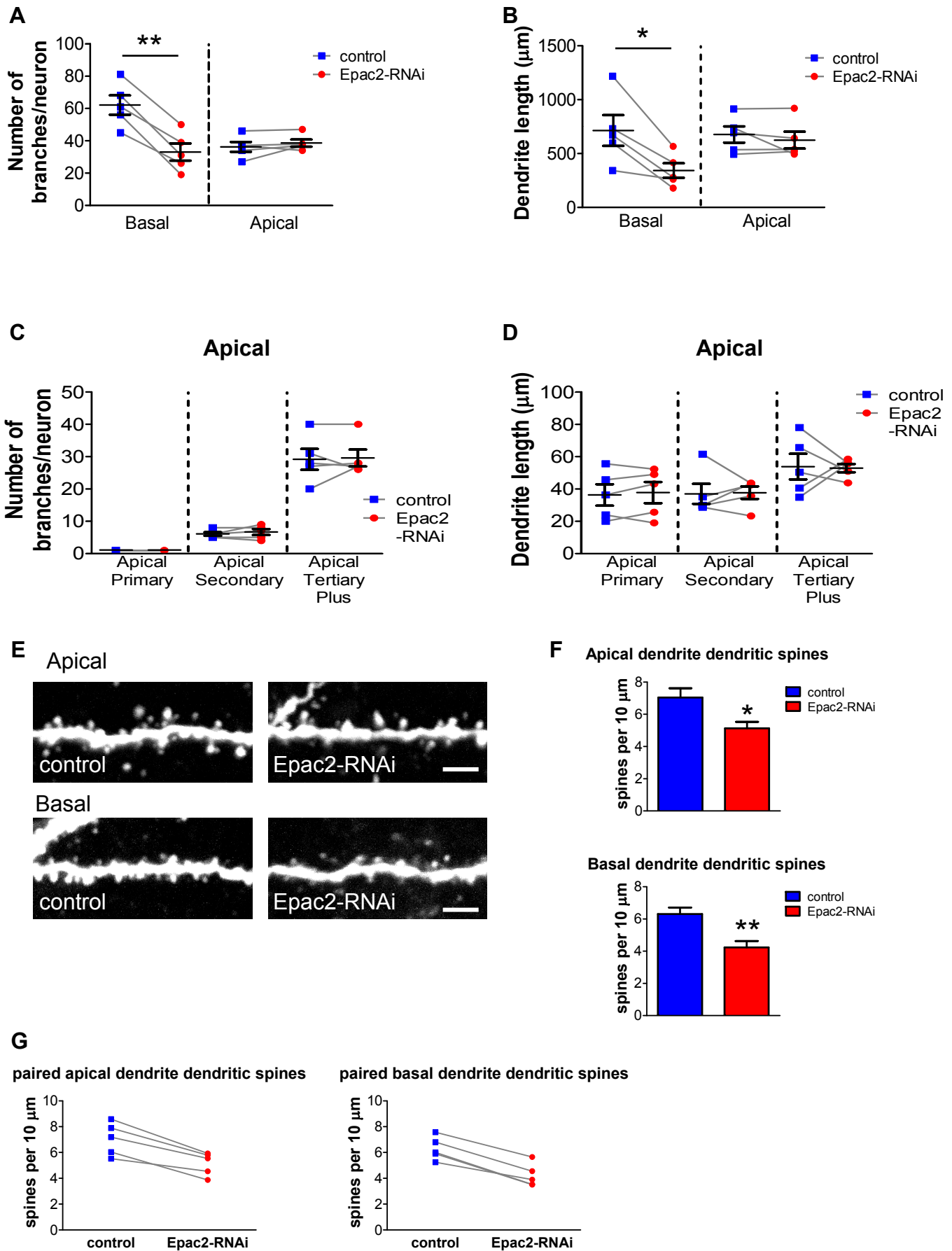

Figure S2

Supplement: Figure S2 — Epac2 regulates higher order basal dendrite complexity, without affecting apical dendrite complexity in vivo. (Related to Figure 2) (A–B) Quantification of total apical and basal dendrite number (A) and length (B) in paired cells; blue square, control; red circles, Epac2-RNAi; black squares with error bars, mean, SEM. (C) Quantification of apical dendrite numbers, separated into primary, secondary, or tertiary and above, reveals no effect of Epac2 knockdown in vivo on apical dendrite number compared to paired control; blue square, control; red circles, Epac2-RNAi; black squares with error bars, mean, SEM. (D) Analysis of apical dendrites, separated into primary, secondary, or tertiary and above, demonstrates no effect of Epac2 knockdown on apical dendrite length. (E) Representative images of dendritic spines in tertiary apical or secondary basal dendrites in control or Epac2-RNAi-expressing layer 2/3 cortical neurons. (F) Quantification of dendritic spine number in tertiary apical or secondary basal dendrites in control or Epac2-RNAi-expressing layer 2/3 cortical neurons. Long-term expression of Epac2-RNAi significantly reduces dendritic spine number in apical and basal dendrites. (G) Epac2-RNAi reduces dendritic spine density on both apical and basal dendrites with pairs of electroporated neurons. *p<0.05, **p<0.01; scale bar, 5 µm. (PDF) [file pbio.1001350.s002.pdf]

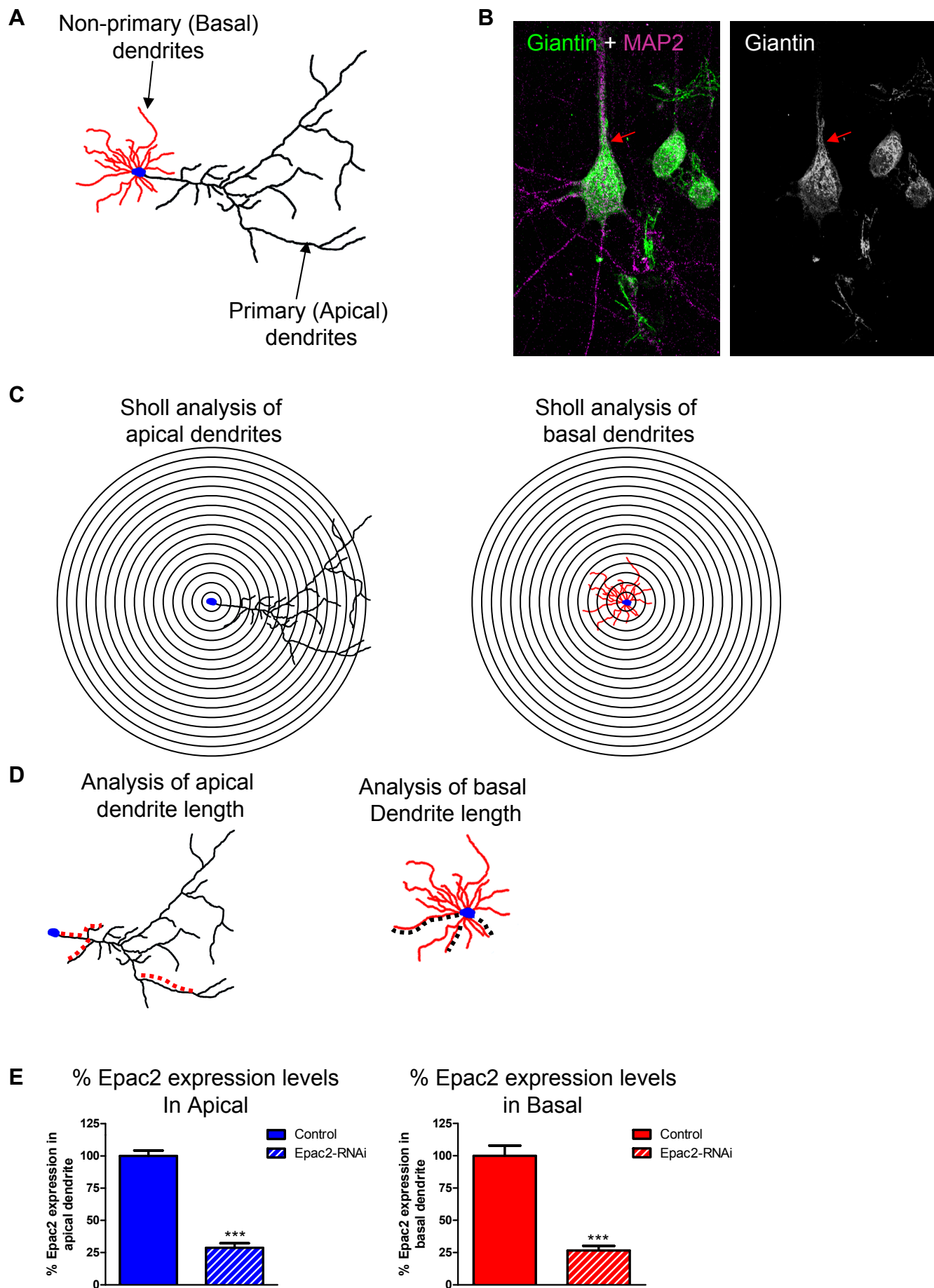

Figure S3

Supplement: Figure S3 — Quantification of dendritic complexity and length. (Related to Figure 3) (A) Representative image of primary, classified as apical (black) and non-primary, classified as basal (red) dendrites of cortical pyramidal neurons (DIV 25). (B) Double immunostaining with MAP2 and Golgi complex marker, giantin. Primary (apical) dendrite is identified by red arrow. Note the presence of Golgi in the apical dendrite, as well as differences between apical and basal dendrite diameters. (C) Schematic of Sholl analysis of basal and apical dendrites separately. (D) Schematic of how dendrite length measurements were performed. Each dendrite was traced (red dotted line represents tracing of apical dendrites, whereas black dotted line represents tracing of basal dendrites), and total dendritic length of each compartment was calculated. (E) Quantification of Epac2 expression levels in apical or basal dendrites in either control (pGSuper) or Epac2-RNAi-expressing cells. In both compartments, Epac2 is knocked down with equal efficiency (∼75%). ***p<0.001. (PDF) [file pbio.1001350.s003.pdf]

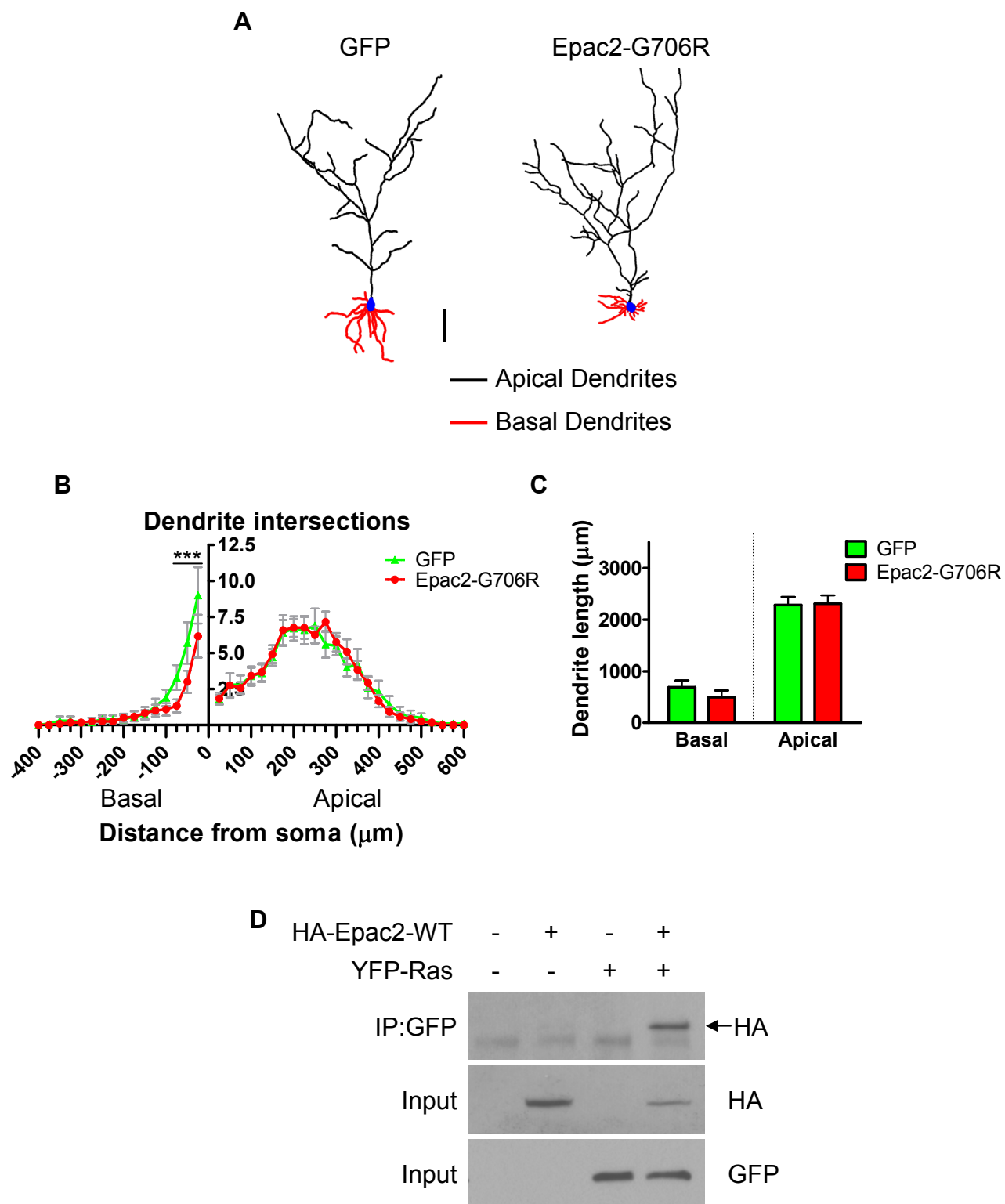

Figure S4

Supplement: Figure S4 — Epac2-G706R overexpression impairs basal dendrite maintenance and interaction with Ras. (Related to Figure 4) (A) Representative binary traces of cortical neurons expressing GFP or GFP+Epac2-G706R. (B) Sholl analysis reveals that complexity of the basal dendrite arbor was reduced in cells expressing Epac2-G706R compared to cells expressing GFP. (C) Quantification of apical and basal dendrite length reveals no difference in dendritic length of basal arbors. (D) Epac2 and Ras coimmunoprecipitate in hEK293 cells. HA-tagged Epac2-WT and YFP-tagged Ras were expressed in hEK293 cells, and immunoprecipitated using a rabbit anti-GFP polyclonal antibody. ***p<0.001; scale bar, 100 µm. (PDF) [file pbio.1001350.s004.pdf]

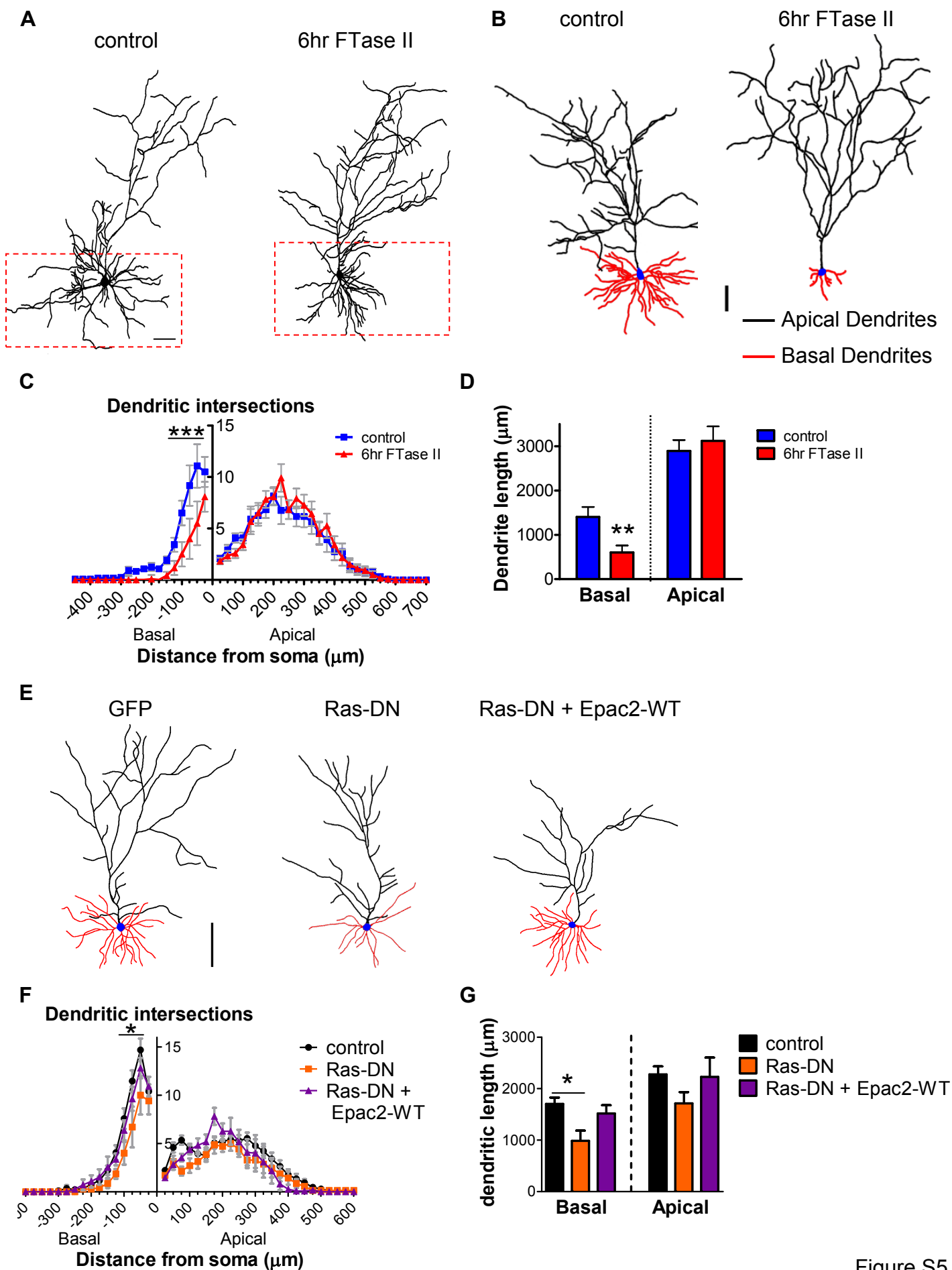

Figure S5

Supplement: Figure S5 — Inhibition of Ras activity reduces basal dendritic maintenance. (Related to Figure 5) (A) Binary images of full dendritic arbors of cortical neurons shown in Figure 5A (dashed red rectangle indicates basal arbor shown in Figure 5A) at −2 hr pretreatment. Neurons were imaged live and treated with vehicle or farnesyltransferase inhibitor II (FTaseII, 200 nM) for 6 h. (B) Binary images of cortical neurons treated with FTaseII (200 nM) or vehicle for 6 h, and subsequently fixed and imaged. (C) Sholl analysis of apical and basal dendrites reveals that FTaseII treatment for 6 h selectively reduces complexity of basal arbors. (D) Quantification of mean dendritic length for apical dendrites and basal dendrites demonstrates a reduction of basal dendrite length induced by FTaseII treatment. (E) Representative binary images of cortical neurons expressing GFP, GFP+RasDN, or GFP+RasDN+Epac2-WT. (F) Sholl analysis of apical and basal dendrites reveals a significant decrease in basal complexity in RasDN-expressing cells; this effect is rescued by Epac2-WT overexpression. (G) Quantification of apical and basal dendrite length. *p<0.05, **p<0.01, ***p<0.001; scale bars, 100 µm (A, B, E). (PDF) [file pbio.1001350.s005.pdf]

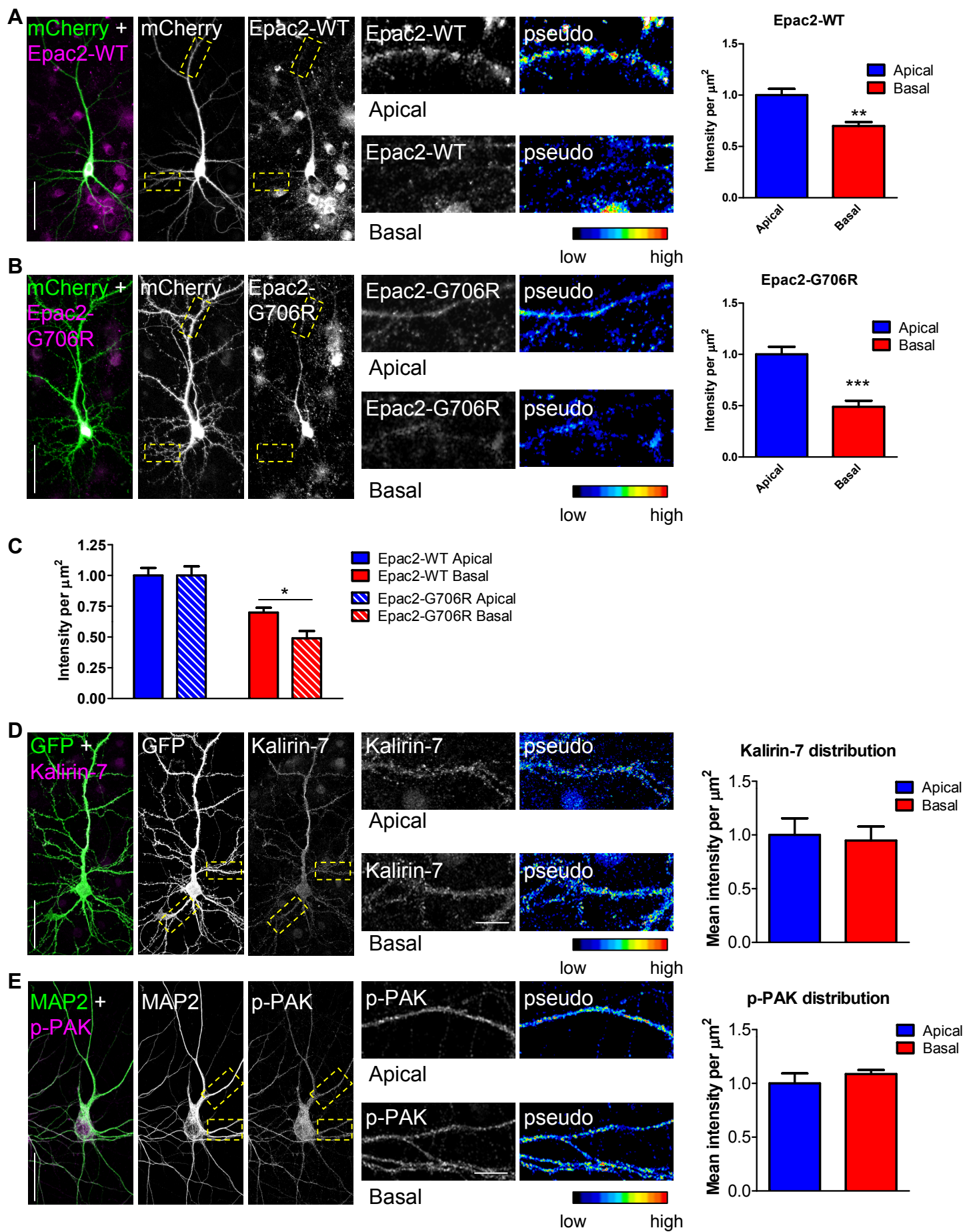

Figure S6

Supplement: Figure S6 — Asymmetric distribution of ectopically expressed Epac2-WT and Epac2-G706R, but lack of asymmetric distribution of kalirin-7 and p-PAK in cortical neuron dendrites. (Related to Figure 6) (A) Distribution of HA-Epac2-WT in apical and basal dendrites. Exogenous mCherry indicates dendrites. HA-Epac2-WT fluorescence intensity (normalized to unit area, µm2, and indicated by pseudocoloring) is reduced in secondary basal dendrites. (B) Distribution of HA-Epac2-G706R in apical and basal dendrites. Exogenous mCherry indicates dendrites. HA-Epac2-G706R fluorescence intensity (normalized to unit area, µm2, and indicated by pseudocoloring) is reduced in secondary basal dendrites. (C) Comparison of Epac2 immunofluorescence levels in (A–B) normalized to Epac2-WT apical levels reveals a significant decrease in basal Epac2-G706R intensity compared to basal Epac2-WT intensity. (D) Distribution of the Rac-GEF kalirin-7 over apical and basal dendrites. Yellow dashed boxes indicate areas in high magnification to the right. Overexpressed GFP was used to outline dendrite morphology. Intensity of kalirin-7 staining was normalized to unit area (µm2) over a 50 µm length of secondary apical or basal dendrite to account for thickness of dendrite. Kalirin-7 fluorescence intensity is equally abundant in basal and apical dendrites. (E) Examination of p-PAK (an indicator of Rac signaling): immunostaining revealed that p-PAK fluorescence intensity is equally abundant in basal and apical dendrites. Endogenous MAP2 was used to indicate dendrites. Scale bars, 35 µm (A, B, D, E), 5 µm (A, B, D, E insets). (PDF) [file pbio.1001350.s006.pdf]

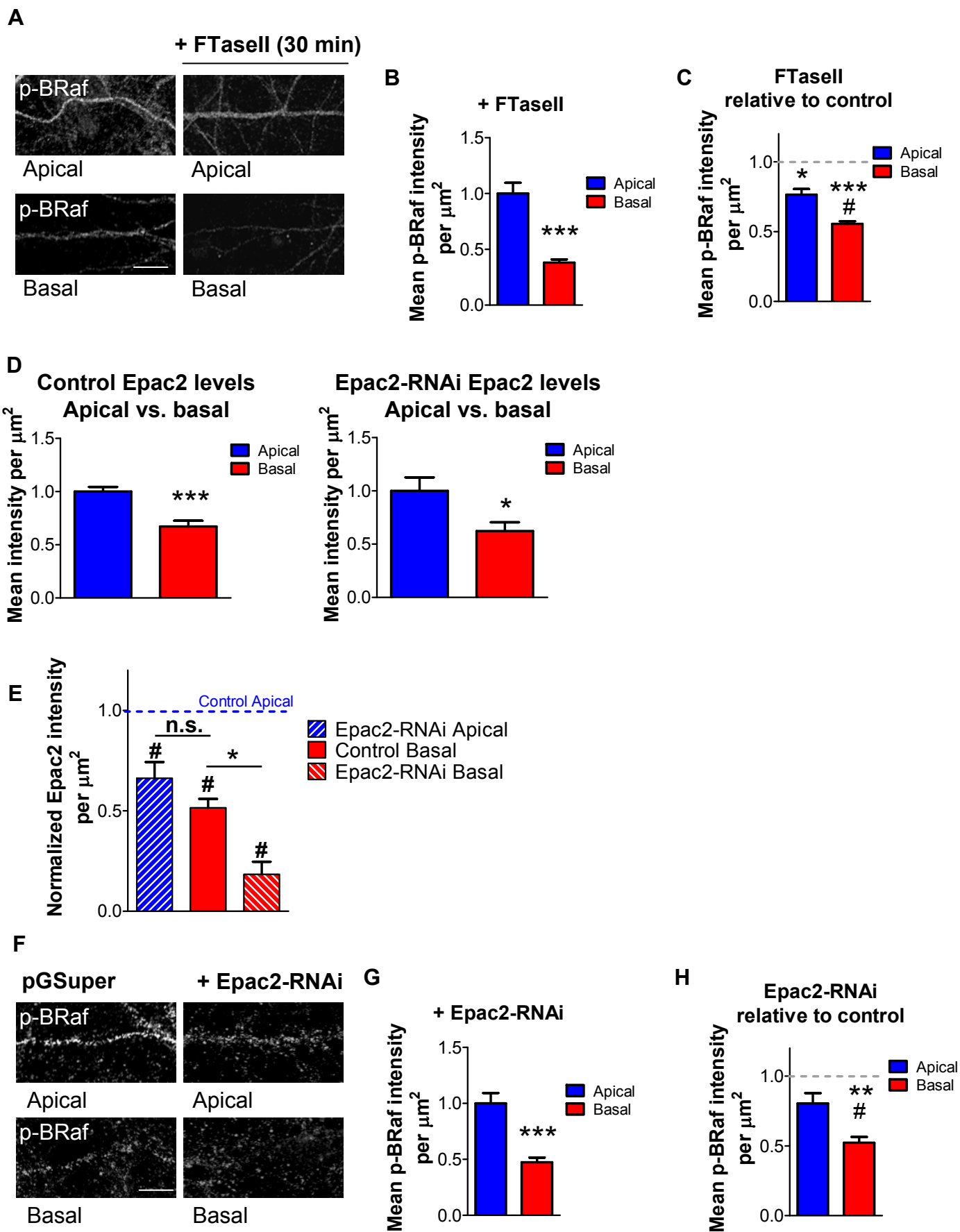

Figure S7

Supplement: Figure S7 — Asymmetric distribution and regulation of p-BRaf in cortical neuron dendrites. (Related to Figure 7) (A) Staining of endogenous p-BRaf in apical and basal secondary dendrites treated with vehicle or FTaseII. Images are high magnifications of areas in yellow dashed boxes indicated in Figure 7A. Inhibition of Ras signaling by FTaseII (30 min, 200 nM) reduced p-BRaf immunofluorescence in basal dendrites compared to apical dendrites. Endogenous MAP2 was used to indicate dendrites. (B) Quantification of p-BRaf immunofluorescence in basal versus apical dendrites following FTaseII treatment; p-BRaf fluorescence intensity was normalized to unit area, µm2. (C) Comparison of p-BRaf immunofluorescence in basal versus apical dendrites after 30 min FTaseII treatment relative to control apical levels (dashed gray line). (D) Quantification of Epac2 distribution in secondary apical or basal dendrites from cells expressing pGSuper (control RNAi) or Epac2-RNAi; Epac2 intensity was normalized to unit area, µm2. Epac2 is asymmetrically distributed with less expression in basal dendrites compared to apical dendrites in both control and Epac2-RNAi expressing cells. (E) Comparison of endogenous Epac2 fluorescence intensities in (D), normalized to control apical levels, reveals a significant decrease in Epac2 levels in control basal dendrites, but no significant difference in Epac2 levels in Epac2-RNAi apical dendrites versus control basal dendrites. (F) Staining of endogenous p-BRaf in apical and basal dendrites in the presence of pGSuper or Epac2-RNAi. Images are high magnifications of areas in yellow dashed boxes indicated in Figure 7D. Inhibition of Epac2 function by Epac2-RNAi resulted in selective and robust reduction of p-BRaf immunofluorescence in basal dendrites compared to apical dendrites. Overexpressed GFP was used to outline dendrite morphology. (G) Quantification of p-BRaf immunofluorescence in basal versus apical dendrites in Epac2-RNAi-expressing cells; p-BRaf fluorescence [file pbio.1001350.s007.pdf]
