# Supplementary material for: An Autism-Associated Variant of Epac2 Reveals a Role for Ras/Epac2 Signaling in Controlling Basal Dendrite Maintenance in Mice
Source: PLoS Biol. 2012 Jun 26;10(6):e1001350. doi: 10.1371/journal.pbio.1001350 (PMC3383751; doi:10.1371/journal.pbio.1001350)
Supplement: Table S1 — Quantification of dendritic morphology for apical and basal dendritic branch number and length in in utero electroporated 50 µm sections. (PDF) [file pbio.1001350.s008.pdf]

Table S1.

A

Quantification of dendrite morphology for apical and basal dendritic branch number in *in utero* electroporated 50 µm sections.								
**Experimental condition**	**Number of branches per neuron (mean ± s.e.m.)**							
	**Basal**				**Apical**			
	Total	Primary	Secondary	Tertiary	Total	Primary	Secondary	Tertiary
**control**	20.3 ± 1.4	5.3 ± 0.5	7.2 ± 0.6	8.5 ± 0.7	16.1 ± 1.6	1.1 ± 0.08	6.2 ± 0.7	8.7 ± 0.7
**Epac2-RNAi**	**11.7 ± 1.04**	4.3 ± 0.4	**5.1 ± 0.5**	**2.8 ± 0.5**	14.0 ± 0.9	1.1 ± 0.09	6.1 ± 0.3	7.9 ± 0.7

B

Quantification of dendrite morphology for apical and basal dendritic branch length in *in utero* electroporated 50 µm sections.								
**Experimental condition**	**Dendritic length (µm) (mean ± s.e.m.)**							
	**Basal**				**Apical**			
	Total dendritic length (µm)	**Average branch length (µm)**			Total dendritic length (µm)	**Average branch length (µm)**		
		Primary	Secondary	Tertiary		Primary	Secondary	Tertiary
**control**	596.3 ± 48.2	19.8 ± 2.8	31.3 ± 2.6	35.7 ± 3.8	508.4 ± 38.5	59.5 ± 5.0	31.6 ± 3.1	30.8 ± 2.2
**Epac2-RNAi**	**269.9 ± 20.6**	22.9 ± 2.6	**23.8 ± 2.4**	**16.1 ± 2.2**	484.8 ± 33.7	67.2 ± 10.2	40.4 ± 4.6	29.9 ± 2.2
