# Supplementary material for: An Autism-Associated Variant of Epac2 Reveals a Role for Ras/Epac2 Signaling in Controlling Basal Dendrite Maintenance in Mice
Source: PLoS Biol. 2012 Jun 26;10(6):e1001350. doi: 10.1371/journal.pbio.1001350 (PMC3383751; doi:10.1371/journal.pbio.1001350)
Supplement: Table S2 — Quantification of apical and basal dendritic branch number in in utero electroporated paired neurons in 300 µm sections. (PDF) [file pbio.1001350.s009.pdf]

Table S2.

Quantification of apical and basal dendritic branch number in *in utero* electroporated paired neurons in 300 µm sections.								
	Number of branches per neuron							
	Basal				Apical			
**Individual Paired cell data**	Total branch number	Number of branches per neuron			Total branch number	Number of branches per neuron		
		Primary	Secondary	Tertiary		Primary	Secondary	Tertiary
control	45	4	17	24	37	1	8	28
Epac2-RNAi	26	4	12	12	38	1	8	27
control	68	8	10	50	34	1	6	27
Epac2-RNAi	31	7	8	17	37	1	7	28
control	81	6	28	47	27	1	6	20
Epac2-RNAi	50	5	18	27	37	1	9	27
control	56	4	12	40	37	1	5	31
Epac2-RNAi	19	3	6	12	34	1	5	26
control	61	6	11	44	46	1	5	40
Epac2-RNAi	39	8	10	23	47	1	4	40

**Combined data**	Average number of branches (mean ± s.e.m)							
	Basal				Apical			
	Average total branch number	Average number of branches			Average total branch number	Average number of branches		
		Primary	Secondary	Tertiary		Primary	Secondary	Tertiary
control	62.2 ± 6.0	5.6 ± 0.7	15.6 ± 3.3	41 ± 4.6	36.2 ± 3.1	1 ± 0	6 ± 0.5	29.2 ± 3.2
Epac2-RNAi	**33 ± 5.4**	5.4 ± 0.9	**10.8 ± 2.1**	**18.2 ± 2.9**	38.6 ± 2.2	1 ± 0	6.6 ± 0.9	29.6 ± 2.6
