# Supplementary material for: An Autism-Associated Variant of Epac2 Reveals a Role for Ras/Epac2 Signaling in Controlling Basal Dendrite Maintenance in Mice
Source: PLoS Biol. 2012 Jun 26;10(6):e1001350. doi: 10.1371/journal.pbio.1001350 (PMC3383751; doi:10.1371/journal.pbio.1001350)
Supplement: Table S3 — Quantification of apical and basal dendritic branch length in in utero electroporated paired neurons in 300 µm sections. (PDF) [file pbio.1001350.s010.pdf]

Table S3.

Quantification of apical and basal dendritic branch length in *in utero* electroporated paired neurons in 300 µm sections.								
	Dendritic length (µm)							
	**Basal**				**Apical**			
**Individual Paired cell data**	**Total dendritic length per neuron**	**Average branch length per neuron**			**Total dendritic length per neuron**	**Average branch length per neuron**		
		Primary	Secondary	Tertiary		Primary	Secondary	Tertiary
**control**	343.5	25.1	27.0	27.3	694.2	55.6	29.9	40.5
**Epac2-RNAi**	261.9	29.8	22.3	16.0	641.7	52.3	43.6	58.4
**control**	598.7	13.4	27.6	34.3	536.2	23.9	35.1	34.9
**Epac2-RNAi**	179.4	11.3	21.7	17.7	539.2	19.0	42.6	55.7
**control**	1218.3	25.8	55.4	54.4	493.3	45.7	61.5	65.6
**Epac2-RNAi**	566.6	18.9	35.2	39.7	518.8	49.1	43.4	50.9
**control**	670.5	25.9	46.3	42.2	737.9	20.1	28.8	78.0
**Epac2-RNAi**	279.9	32.2	35.5	30.7	494.9	25.6	23.3	55.9
**control**	731.3	15.8	48.4	55.3	914.7	36.4	29.7	50.4
**Epac2-RNAi**	415.3	16.9	37.6	41.3	921.1	43.1	35.7	43.8

	Average dendritic length (µm) (mean ± s.e.m)							
	**Basal**				**Apical**			
**Combined data**	**Average Total dendritic length**	**Average branch length (µm)**			**Average Total dendritic length**	**Average branch length (µm)**		
		Primary	Secondary	Tertiary		Primary	Secondary	Tertiary
**control**	712.4 ± 142.7	21.2 ± 2.7	40.9 ± 5.8	42.7 ± 5.5	675.3 ± 75.5	36.3 ± 6.6	37.0 ± 6.2	53.9 ± 7.9
**Epac2-RNAi**	**340.6 ± 68.0**	21.8 ± 3.9	**30.4 ± 3.5**	**29.1 ± 5.3**	623.5 ± 78.6	37.8 ± 6.5	37.7 ± 3.9	52.9 ± 2.6
